# Assessment of urban greenhouse gas emissions towards reduction planning and low-carbon city: a case study of Montreal, Canada

**DOI:** 10.1186/s40068-024-00341-y

**Published:** 2024-04-16

**Authors:** Shadnoush Pashaei, Chunjiang An

**Affiliations:** Department of Building, Civil and Environmental Engineering, Concordia University, Montreal, H3G 1M8 Canada

**Keywords:** Greenhouse gas, Emission assessment, Urban activities, Sensitivity analysis

## Abstract

Greenhouse gases (GHGs) can be produced from a broad range of anthropogenic activities at different spatial and temporal scales. In particular, emissions from urban area are an import source of GHGs. City is a complicated system consisting of various component and processes. Efforts have been made to reduce urban GHG emissions. However, there is a lack of available methods for effective assessment of such emissions. Many urban sources and factors which can influence the emissions are still unknown. In the present study, the GHG emissions from municipal activities was assessed. A model for the assessment of urban GHG emissions was developed. Based on the collected data, a case study was conducted to evaluate urban GHG emissions. The comprehensive assessment included the emissions from transportation, electricity consumption, natural gas, waste disposal, and wastewater treatment. There was a variation for GHG emissions from these sectors in different years. This study provided a new approach for comprehensive evaluation of urban GHG emissions. The results can help better understand the emission process and identify the major emission sources.

**Supplementary Information:**

The online version contains supplementary material available at 10.1186/s40068-024-00341-y.

## Introduction

There is a growing concern for earth temperature increase caused by anthropogenic perturbation (Samaniego et al. 2018). The rising greenhouse gas (GHG) emissions result in the change of radiative pattern in atmosphere, which would increase the average surface temperature and eventually lead to the changing global climate (Opio et al. 2023). GHGs can be produced from a broad range of anthropogenic activities at different spatial and temporal scales (Biramo and Mekonnen 2022; Chen et al. 2020). In particular, emissions from urban areas are an import source of GHGs (Tian et al. 2020; Yu et al. 2017). 54% of the global population lived in urban areas in 2014 and by 2050, this ratio will increase to 66% of the global population. About 75% of energy consumption and 80% of GHG emissions globally can be attributed to the urban activities (Hu et al. 2016). Cities may consume a large amount of energy to meet the demands of transport, industrial and commercial, heating and cooling activities. In addition, solid wastes and wastewater are also mostly produced in urban agglomerations (Ebner et al. 2015). Therefore, the efforts of municipalities are crucial for achieving the goal of GHG reduction (Zhang et al. 2007).

GHG inventory is a tool to evaluate the status of emissions and the potential for mitigation. The Intergovernmental Panel on Climate Change (IPCC) provides a detailed methodological framework to accomplish the inventories (IPCC 2006). It assesses the greenhouse gases emitted from main sectors including energy, industrial processes and product use, agriculture, forestry and other land use, and waste. These emission inventories provide a general picture of large-scale patterns of greenhouse gas emissions. City is a complicated system consisting of various components and processes (National Environmental Research Institute 2005). Some studies about the GHG emissions assessment in urban areas have been reported previously. Qi et al. (2018) investigated the inventory of GHG emissions and its environmental and economic impacts on Jinan, using a hybrid Life Cycle Assessment (LCA) method. The economic burden on human health was also compared with that on GHG emissions and ecosystem. Gurjar et al. (2004) reported an emission inventory for Delhi, including a range of air pollutants and GHG emissions. Power plants were found to be the main emission source of SO2 and suspended particles, while the transport sector was the largest source of NOx, CO and non-methane volatile organic compound. Hillman and Ramaswami (2010) proposed a hybrid life cycle-based GHG emission assessment method for cities. The cross-boundary activities were found to be an important contributor to urban GHG emissions. Schmidt Dubeux and Rovere (2007) studied the GHG emissions of Rio de Jarneiro and the potential benefits from GHG reduction measures were evaluated. In addition, some municipalities also conducted some general GHG emission assessments (Environment and Energy Program Administration, 2018; Oslo, 2018; York, 2017). In addition, digital twin simulation has also been used to assess GHG emissions in smart cities. However, there is still a lack of available methods for effective assessment of such urban emissions. Many urban sources and factors which can influence the emissions are still unknown. Therefore, there is an urgent need to determine the urban GHG sources and evaluate the emissions in a comprehensive manner.

In the present study, the GHG emissions from municipal activities will be assessed from a new perspective. A generalized model will be developed for the assessment of urban GHG emissions at first. Based on the collected data of Montreal in Canada, a case study will then be conducted to evaluate the GHG emissions from transportation (i.e. public and private), electricity consumption, natural gas use, waste disposal and wastewater treatment. To better understand the emission patterns, the interaction among different factors in the model will be investigated based on factorial analysis. The uncertainty of modeling will also be determined. The results can help better understand the urban GHG emission characteristics and develop the corresponding strategy for GHG reduction.

## Methodology for urban GHG emission analysis

### Urban GHG emission sources

**Fig. 1:**
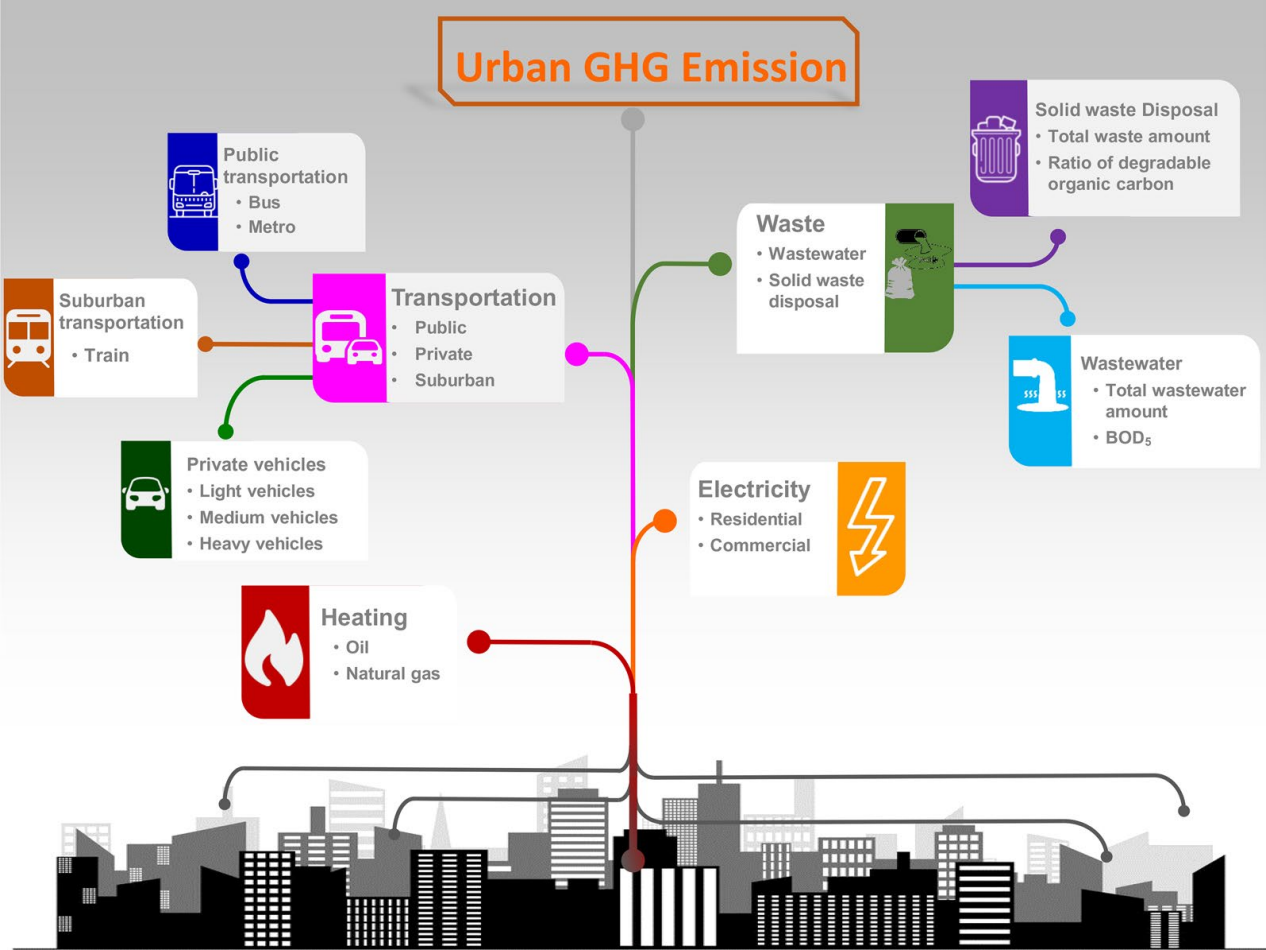
Urban GHG emission sources

Cities play an important role the modern society. GHGs can be derived from many sources in the urban area. As shown in Fig. 1, the main sources of GHGs in urban areas typically include emissions from transportation, heating, electricity generation, and waste processing.

### Emissions from urban public transportation

Public transportation is considered as an effective way to reduce overall GHG emissions, although fossil fuel such as gasoline and natural gas is still consumed in this process. Substantial resources have been used to meet the transportation needs of the urban population. The following has been developed based on different fuels to consider all GHG emissions produced by public transportation. The significant point to apply the below methodology in different fuels is to use the same unit for the amount of fuel consumption as the unit of emission factor. The GHG emissions in this section can be calculated based on the consumption of different energy types.

1 $${{\text{E}}}_{{\text{UPT}}}= \sum {\text{AM}}\_\mathrm{ENERGYi }\times \mathrm{ EF}\_{\text{ENERGYi}}$$

E_UPT: GHG emission from urban public transportation (kg CO_2_-eq).

AM_ENERGY: The amount of energy consumed (GJ).

EF_ENERGY: Emission factor of the energy (kg CO_2_-eq/GJ).

i: Energy types including diesel, gasoline, electricity, biodiesel, etc.

### Emissions from suburban public transportation

In urban areas, there is a suburban public transportation network such as commuter trains to connect urban and suburban areas. They mostly include trains, buses, and taxies which sometimes are neglected in the municipal assessment of GHG emissions, even though their routes pass within the city and this network GHG production should be included in the urban GHG emission assessment (Zahabi et al. 2012). However, they play the main role to mitigate GHG emissions by the reduction of number of private vehicles on roads, burning the enormous amount of fossil fuel should be considered in assessment of GHG emissions.

2 $${\text{E}}\_{\text{SPT}}= \sum ({\text{AWj}},\mathrm{k }\times \mathrm{ TTDj},{\text{k}}) \times \mathrm{ EF}\_\mathrm{ SPTN j}$$

E_SPT: GHG emission from suburban public transportation (kg CO_2_-eq).

AW: Average weight of suburban public transportation vessel (tonne).

TTD: Total travel distance of suburban public transportation vessel (km).

EF_SPTN: Emission factor of suburban public transportation type (kg CO_2_-eq/tonne-km).

j: Suburban public transportation types.

k: Suburban public transportation routes.

### Emissions from private vehicles

One of the largest drivers of urban GHGs emissions is vehicles. As the number of private vehicles on the streets continues to rise, their impact becomes increasingly significant. In this study to clarify the role of each sector, the contribution factors considered as separated as they could, that is why vehicles divided into three parts such as urban public transportation, suburban public transportation, and private vehicles. Moreover, private vehicles categorized into three categories defined by the weight of vehicles such as light vehicles (less than 4500 kg, e.g. cars, vans, light pickups, station wagon, van, SUV, and motorcycles), medium vehicles (between 4500 kg and 9000 kg, e.g. heavy-duty pickups and medium size work trucks), and heavy vehicles (greater than 9000 kg, e.g. garbage trucks and tandem dump trucks) (Australian Transport Assessment and Planning (ATAP) 2016). The different methodologies can be applied in this section. One approach would be based on the number of vehicles and another one based on fossil fuel amount consumed by vehicles. The methodology indicated in this study is according to the number of vehicles registered in the area boundary.

3 $${\text{E}}\_\mathrm{VEH }= \sum ({\text{N}}\_\mathrm{VEHm }\times \mathrm{ ATDm }\times \mathrm{ FE}\_{\text{VEH}}) \times \mathrm{ EF}\_{\text{FUEL}}$$

E_VEH: GHG emission from vehicles (kg CO_2_-eq).

N_VEH: Number of vehicles.

ATD_VEH: Average travel distance per vehicle (km/vehicle).

FE_VEH: Fuel efficiency (L/km).

EF_FUEL: Emission factor of fuel consumption (kg CO_2_-eq /L).

m: Vehicle type.

### Emissions from fuel-based heating

To meet the living requirements, heating provided in urban areas can be one of the most significant contributing factors to urban GHGs emissions. Natural gas is often used as a major fuel type for heating in the municipal area. Burning natural gas to provide heating, produces greenhouse gases such as CO_2_, CH_4,_ and N_2_O. Some other fuel types such as heating oil are also used in some areas as well as electricity, although using electricity with the aim of heating should be considered in the electricity section (OVOenergy 2010). The consumption of these fuels for heating will be associated with the generation of GHGs. The methodology developed in this study can be applied for any kind of fuel consumed for heating-target. The GHGs from heating can be calculated as follows.

4 $${{\text{E}}}_{{\text{HEAT}}}=\sum {\text{AM}}\_\mathrm{HEATn }\times \mathrm{ EF}\_{\text{HEATn}}$$

E_HEAT: GHG emissions from heating (kg CO_2_-eq).

AM_HEAT: Total amount of heating fuel (m^3^ or GJ).

EF_HEAT: Emission factor of heating fuel (kg CO_2_-eq/m^3^ or kg CO_2_-eq/GJ).

n: heating fuel types.

### Emissions from electricity generation

Electricity is extensively consumed for residential, commercial, and industrial activities in urban areas. GHG emission is associated with the generation of electricity, and it can be regarded as the indirect emission for the urban system. The electricity can be produced from power plants using natural gas, coal, fuel oil, geothermal energy, solar power, and hydropower plants, that is why the ratio of electricity generation should be considered in the methodology. Definitely, the greenhouse gas produced by the burning of fossil fuel (coal, natural gases) is the main component in this section and electricity generated by hydropower would have the least contribution ratio to GHGs emissions. The total GHG emission from electricity is as follows.

5 $${\text{E}}\_\mathrm{ELEC }= \sum {\text{AM}}\_{\text{ELECs}}\times {\text{RATIO}}\_{\text{ELECt}}\times {\text{EF}}\_{\text{EGSt}}$$

E_ELEC: GHG emission from electricity (kg CO_2_-eq).

AM_ELEC: Amount of electricity consumption in section (GWh).

RATIO_ELEC: Ratio of electricity generation from different sources.

EF_EGS: Emission factor in electricity generation source (kg CO_2_-eq/GWH).

s: Sections of electricity consumption in residential, institutional, commercial and industrial sectors.

t: Electricity generation sources including hydropower, thermopower, solar power, etc.

### Emissions from solid waste disposal

The disposal and treatment of solid waste can result in the emissions of several GHGs. In this study, the methodology developed to model the waste sector uses the greenhouse gas emissions of the landfill as the main effect (Prakash and Bhat 2012). The major GHG released from this process is CH_4_ and CO_2_ and it is emitted during the breakdown of organic matter from solid waste in the disposal process (Ebner et al. 2015). The GHGs produced from solid waste disposal can be evaluated as follows.

6 $${{\text{E}}}_{{\text{SWD}}}= [({\text{AM}}\_\mathrm{MSW }\times \mathrm{ DOC}\_\mathrm{SW }\times \mathrm{ F}\_\mathrm{DOC }\times \mathrm{ MCF }\times \mathrm{ F}\_\mathrm{MLG }\times 16/12) -\mathrm{ RM}] \times (1 -\mathrm{ OF}) \times \mathrm{ GWP}\_{\text{CH}}4$$

E_SWD: GHG emission from solid waste disposal (kg CO_2_-eq).

AM_MSW: Total amount of municipal solid waste in the year (kg waste).

DOC_SW: Degradable organic carbon in solid waste (kg carbon/kg waste).

F_DOC: Fraction of degradable organic carbon dissimilated.

MCF: Methane correction factor.

F_MLG: Fraction of methane in landfill gas.

16/12: Conversion of C to CH_4_.

RM: Recovered methane (kg CH_4_).

OF: Oxidation factor.

GWP_CH_4_: Global warming potential of CH_4_ (kg CO_2_-eq/kg CH_4_).

### Emissions from wastewater treatment

High removal of Biochemical Oxygen Demand (BOD), Chemical Oxygen Demand (COD), organic carbon, nutrients and pathogenic microorganisms from wastewater can be achieved by WasteWater Treatment Plants (WWTPs). CO_2_ and CH_4_ production results from the breakdown of organic matter in the activated sludge process and some through the primary clarifiers, degradation of nitrogen components in the wastewater also result in N_2_O production (Gupta and Singh 2012). The operation of municipal wastewater treatment plants is also associated with the emission of GHGs. CH_4_ can be produced from wastewater when treated or disposed of anaerobically. N_2_O and CO_2_ emissions can be emitted from wastewater. Reducing these emissions from the treatment process and the contribution of the WWT processes to global warming is a major concern (Listowski et al. 2011). The GHG emission from the wastewater treatment process can be calculated using the following equation.

7 $${\text{E}}\_\mathrm{WT }=\mathrm{ E}\_{\text{WTCH}}4 +{\text{E}}\_{\text{WTN}}2{\text{O}}$$

E_WT: GHG emission from wastewater treatment (kg CO_2_-eq).

E_CH_4_: Emission of CH_4_ (kg CO_2_-eq).

E_N_2_O: Emission of N_2_O (kg CO_2_-eq)

8 $${\text{E}}\_{\text{CH}}4 =\mathrm{ AM}\_\mathrm{WW }\times \mathrm{ CBOD }\times \mathrm{ EF}\_{\text{CH}}4 \times \mathrm{ GWP}\_{\text{CH}}4 \times 10-6$$

AM_WW: Amount of wastewater (L).

CBOD: Concentration of BOD_5_ in wastewater (mg/L).

EF_CH_4_: Emission factor (kg CH_4_/kg BOD_5_)

9 $${\text{E}}\_{\text{N}}2\mathrm{O }=\mathrm{ AM}\_\mathrm{WW }\times \mathrm{ CN }\times \mathrm{ EF}\_{\text{N}}2\mathrm{O }\times \mathrm{ GWP}\_{\text{N}}2\mathrm{O }\times 1.57 \times 10-6$$

CN: Concentration of nitrogen in wastewater (mg N/L).

EF_N_2_O: Emission factor (kg N_2_O-N/kg N).

GWP_N_2_O: Global warming potential of N_2_O (kg CO_2_-eq/kg N_2_O).

1.57: Conversion factor of kg N_2_O-N into kg N_2_O.

### Study area

Montreal is Canada’s second-largest city with the population of about 1.7 million. It is the largest metropolis in the province and is the second-most populous city in Canada for a century and a half. It is located at the confluence of the St. Lawrence and Ottawa rivers and in southwestern Quebec. The land area of Montreal is 365.65 km^2^ and the population density was 4,662.1/km^2^. In 2016, there were 779,802 private dwellings occupied in Montreal (Ville), representing a change of 2.6% from 2011 (Statistics Canada 2019). The emission sources considered in Montreal are transportation, fuel-based heating, electricity, solid waste disposal, and wastewater treatment. In the present study, the boundary of Montreal Island was used for the emission assessment.

## Results and discussion

### Emissions from urban transportation network

Transportation activities has the direct impact on GHG emissions and air quality (Tian et al. 2023a, b; Tian et al. 2023a, b). Montreal is one of Canada’s major metropolitan areas and has a well-developed and well-performing public transit network. Public transportation has been in operation in Montreal for over 150 years. For the present study, the energy consumption of public transportation in Montreal was obtained from Société de Transport de Montréal (STM) with respect to two different sectors, energy consumption by transit stations and vehicles. To avoid any overlaps between electricity and natural gas, only the vehicle sector is considered. In this regard, both Énergir and Hydro-Quebec, the companies responsible for the distribution of natural gas and electricity in Montreal, respectively, have already published data on the usage for major industries such as STM or RTM (suburban transportation by train) in their annual reports (MELCCFP Quebec 2018). The data is categorized by fuel (e.g., diesel, gasoline, natural gas, hydropower, and biodiesel), although Énergir reports that there is no public transport consuming natural gas, meaning that the fuels considered in this sector are diesel, gasoline, hydropower, and biodiesel and the amount these sources of by transportation is represented in Additional file 1: Table S1. The major fuel in the Montreal transportation network is diesel (Société de transport de Montréal STM, 2016). As with other cities in North America, efforts are underway to encourage other technologies such as diesel-electric hybrid bus technology and battery electric bus technology in order to reduce GHG emissions in this sector.

**Fig. 2:**
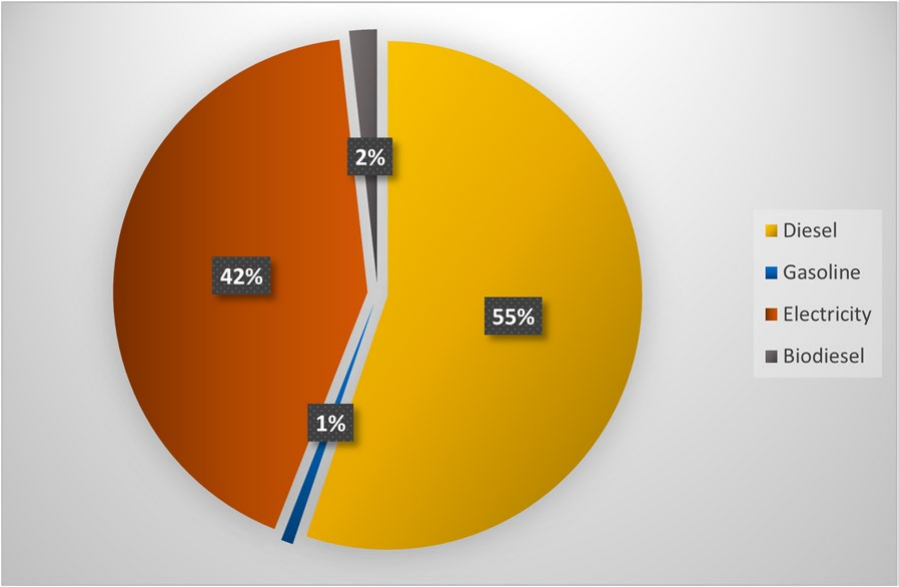
Energy consumption from public transportation in Montreal in 2016

The method developed for this sector is based on fuel consumption by public transportation vehicles such as buses and subway trains (Government of Canada 2017a). The total amount of fuel consumption is multiplied by the emission factor which is drawn from different data sources for different jurisdictions and units, shown in Additional file 1: Table S2. Additional file 1: Table S3 dedicates the amount of energy consumption over 10-year period from 2006 to 2015 in order to figure out the trend of GHG emissions produced by urban transportation network over the period. These energy consumption provided by annual reports of STM (Société de transport de Montréal STM, 2016) have been applied by the developed methodology to obtain GHG emissions. Figure 2 showing the ratios of energy consumption in Montreal in 2016, illustrates the primary source of urban public transportation in the city is diesel by 55% and the second source of energy for this sector is electricity with 42%.

**Fig. 3:**
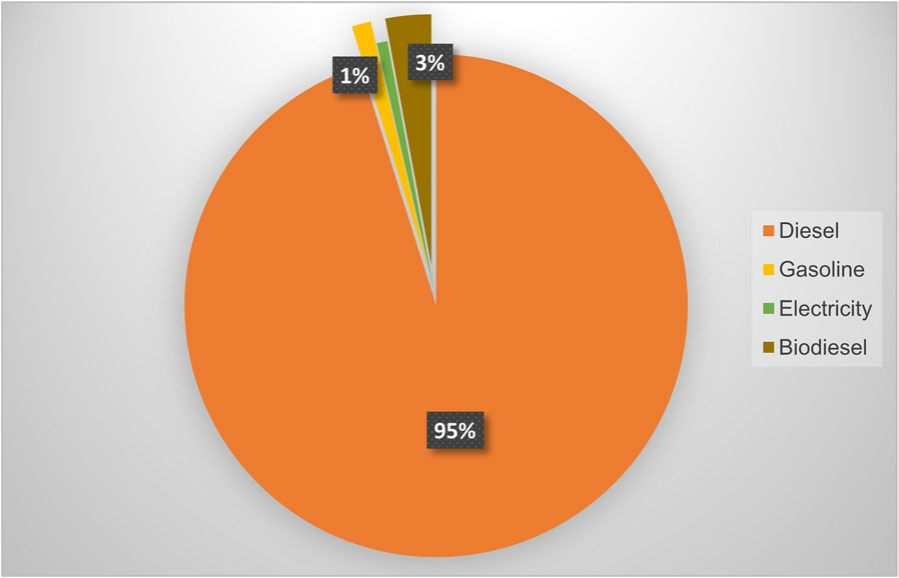
GHG emissions from public transportation in Montreal City by sources in 2016

Electricity in Montreal is produced by two main sources: hydropower and thermal power, with ratios of 99% and 1%, respectively (Hydro-Quebec 2018). Thermal power is described on Hydro-Quebec’s website as primarily representing gasoline and then, diesel. It is for this reason that, in the present study, electricity is estimated separately with different ratios and emission factors for thermal power (primary gasoline) versus hydropower employing an emission factor of 69.3 kg CO_2_-eq/GJ for the former and an emission factor of zero for the latter (Hillman and Ramaswami 2010; Agência Portuguesa do Ambiente, 2017). In most studies of this nature, it should be noted, the emission factor for hydropower considered for the LCA is 0.025 kg CO_2_-eq/kWh while the operation emission factor is 0 (William Steinhurst and Schultz 2012). Since the present study concentrates on urban GHG emissions and the estimation spans a single year (2016), zero is the standard emission factor considered here. Ostensibly, the largest share of GHG emissions from electricity is attributable to thermal power. Figure 3 shows that in 2016 diesel corresponded to emissions of 138.65 million kg CO_2_-eq to the atmosphere, representing the highest GHG emissions among fuels by %95, used in public transportation in Montreal at 95%. Meanwhile, electricity is the second-largest fuel in Montreal as per a 2016 study, representing 42% of total energy consumption. Interestingly, only 993.77 t CO_2_-eq in emissions was produced by electricity generated by thermal power, owing to the low rate of electricity generation of this source. Biodiesel and gasoline, with rates of 2% and 1%, respectively, are the other fuels consumed in this sector. Despite the low rate of biodiesel, it is the second-largest driver of GHG emissions in Montreal Island at 3% of total emissions, while gasoline was the second-least significant driver of GHG emissions by the public transportation at 1%.

**Fig. 4:**
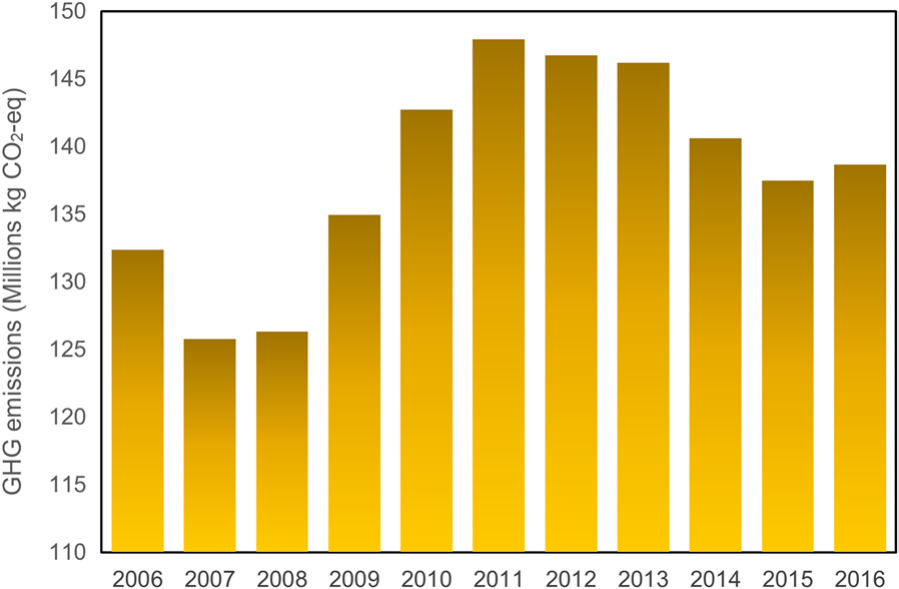
GHG emissions from diesel consumption in public transportation

Figure 4 illustrates the GHG emissions by diesel during the period 2006 to 2016, showing a sudden reduction in 2007 and then remaining at that level in 2008. This was followed by an increase from 125 kt CO_2_-eq in 2008 to about 135 kt CO_2_-eq in 2009, peaking in 2011 at approximately 149 kt CO_2_-eq. It had decreased slightly by 2015, while by 2016 it had seen slight growth. It is now anticipated to remain at a constant level, as the Quebec government is seeking to encourage the use of hybrid buses.

**Fig. 5:**
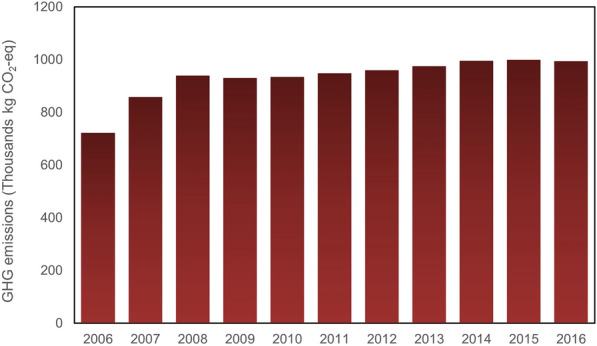
GHG emissions from electricity consumption in public transportation

**Fig. 6:**
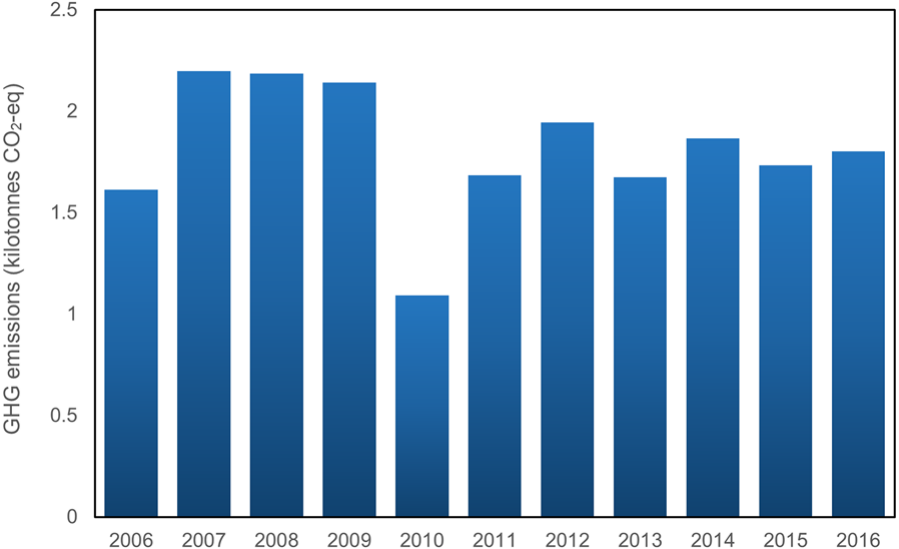
GHG emissions from gasoline consumption in public transportation

As discussed above, electricity is generated by two sources, hydropower and thermal power which present the same trend of consumption over the 10-year period from 2006 to 2016 shown by Figs. 5 and 6, although the GHG emissions produced by these two different sources are different due to their emission factors. The emission factor for electricity generated by hydropower is zero (Koffi et al. 2017) which makes the GHG emissions by electricity generated by hydropower zero. So that, the GHG emissions produced by this source only belongs to electricity generated by thermal power sources. At the beginning of the period, the trend experienced sudden growth and then continued to rise gradually up to 2014, remaining stable until 2016. Due to the low rate of electricity generation, the GHG emissions produced by electricity generated by thermal power are very low, representing emissions of 993.76 kg CO_2_-eq, although the amount of CO_2_-eq produced by gasoline is high.

**Fig. 7:**
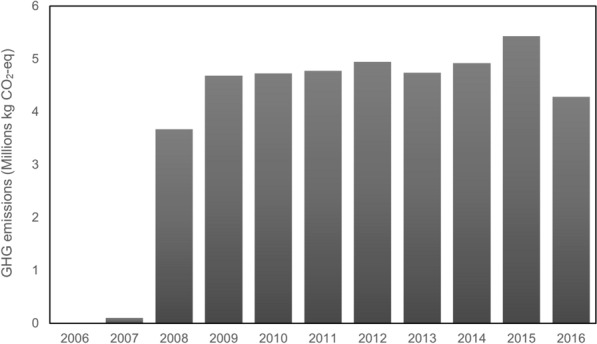
GHG emissions from biodiesel consumption in public transportation

Figure 7 illustrates GHG emission by biodiesel consumption in the urban transportation network (there is no data for biodiesel in 2006.) As can be seen, biodiesel emissions saw a sudden growth in 2008, had stabilized by 2015, then decreased moderately in 2016, with this increase corresponding to an increase in the use of electricity as a fuel source. The fuel consumption data is provided in the STM annual reports, although the amount of biodiesel consumption for not specified in the 2006 report. The GHG emissions produced by biodiesel in 2007 were very low, although it had increased to approximately 4 kt CO_2_-eq by 2008 and then saw a sudden growth in 2009. It had stabilized by 2011, undergoing some changes between the years 2012 and 2014, and then peaking in 2015 with more than 5 kt CO_2_-eq. The figure shows a sudden reduction in 2016, indicative of the efforts of the government to encourage use of hybrid buses.

### Emissions from suburban transportation network

**Fig. 8:**
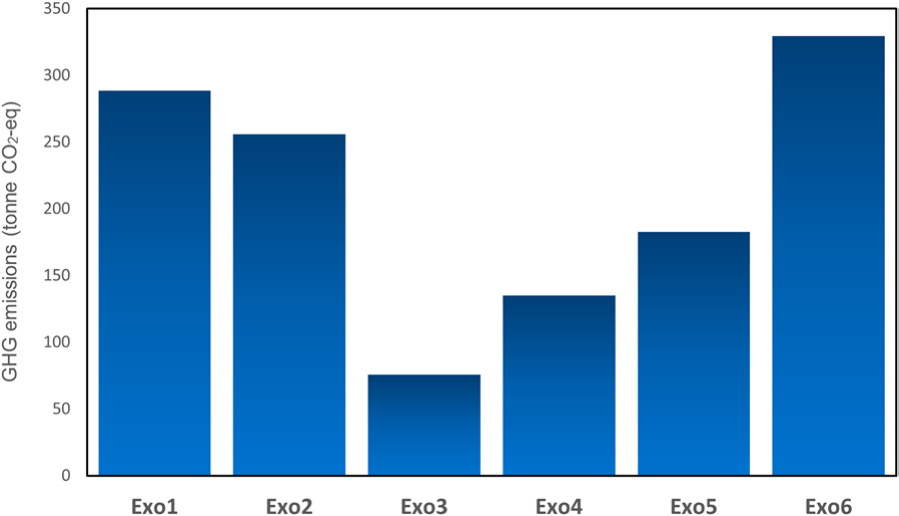
GHG emissions from suburban transportation network in 2016

The Island of Montreal is surrounded by other small islands and cities, resulting in a large population of people commuting to Montreal on a daily basis. Régie des Transports Métropolitains (RTM) refers to the lines that move the passengers by commuter train. In Montreal, there are 6 lines: Exo1-Vaudreuil–Hudson, Exo2-Saint-Jérôme, Exo3-Mont-Saint-Hilaire, Exo4-Candiac, Exo5-Mascouche, and Exo6-Deux-Montagnes. Due to the linking of Montreal proper to the suburbs by these six lines, in the current study, these lines are referred to as the Suburban transportation Network (STN), and the estimation is carried out according to the total distance traveled by these trains within Montreal. Due to the lack of data about the distance traveled by trains, this distance was obtained by checking the distances on the train maps available on the Exo Quebec website (EXO 2019), then multiplying this by the number of shifts per day and then by 252 days excluding weekends and holidays which no services offer in a year to achieve the total distance traveled by these trains annually (Weather Network 2020). Accordingly, Exo1-Vaudreuil–Hudson is found to have traveled 160,855.5 km, Exo2-Saint-Jérôme to have traveled 142,644.19 km, and Exo3, 4, 5, and 6 to have traveled 42,157.5 km, 75,193.65 km, 101,966.4 km, and 183,806.7 km, respectively. The emission factor for these trains is 0.0152 kg CO_2_-eq/tonne-km (CN 2020). The average weight of the trains is another factor considered in the calculation. Due to the fact that different trains are driven in each line, after finding the types of trains and their weights, the average train weight is determined for use in the emission calculations. Therefore, the only variable factor is the travel distance in each line, which is why as shown in Fig. 8, the highest GHG emission belongs to Exo-6, with the longest distance. This line, in particular, was found to have emitted 329.43 t CO_2_-eq in 2016, while the total GHG emissions produced by the STN in Montreal in 2016 was 1,266.47 t CO_2_-eq. However, the second-largest GHG emitter was Exo 1 (Vaudreuil-Hudson), traveling 160,855.5 km by 288.30 t CO_2_-eq. Exo 3 traveled the least distance at 42,157.5 km, in turn accounts for the least GHG emissions at 75.55 t CO_2_-eq.

### Emissions from private vehicles

The methodology typically used for quantifying GHG emissions by the transportation sector is either fuel-based or vehicle-based, where the method employed depends on the activity data available (GHG Emissions Protocol 2016). For the present study, a methodology was developed based on the number of vehicles registered in a given year in the urban area under study. Statistics on registered vehicles were obtained from Société de l’assurance automobile Québec (SAAQ) and Additional file 1: Table S4 shows the number of vehicles in circulation in Montreal from 2002 to 2016 (Societe de Anssurance Automobile Québec, 2007, 2008, 2009, 2016). The data was collected during the registration of road vehicles, where the number of vehicles considered to be in circulation corresponds to the number of vehicles whose license plate is current as of December 31 of the given year. The vehicles in storage, decommissioned, or otherwise unregistered before that date, therefore, are not included in the total. In addition, trailers and vehicles used exclusively at railway stations, ports and airports are not considered in the calculation of the number of vehicles in circulation. The vehicles are grouped according to the category of use (U.S. EPA 2018). Additional file 1: Table S5 shows the number of registered vehicles in Montreal Island based on the categorization of the present study. As can be seen in the table, under the definition for medium vehicles there is no number. For this reason, the estimation was limited to light and heavy vehicles. The numbers for registered vehicles are extracted from the annual reports; due to the lack of an annual report for 2010, it should be noted, there is no information for this year. The methodology developed for private vehicles includes 4 factors: number of vehicles registered in a given year, average travel distance by each type of vehicle, fuel economy of each type of vehicle, obtained from the Transport Canada website (Transport Canada 2019), and emission factor for each type of vehicle. The emission factors based on the different types of vehicles, sources, units, and locations are provided in Additional file 1: Table S6.

**Fig. 9:**
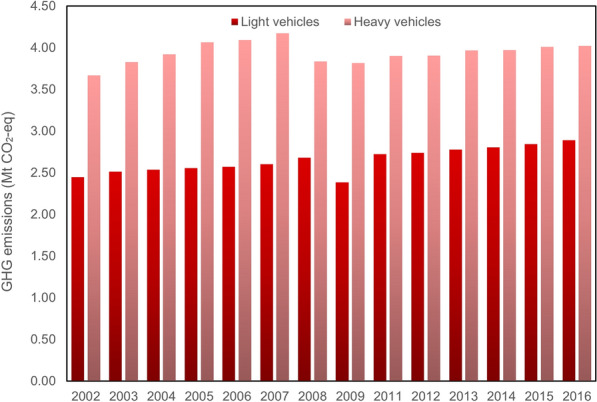
GHG emissions from vehicles

Figure 9 shows the GHG emissions from private vehicles over the period of 2002 to 2016. GHG emissions produced by heavy vehicles in Montreal are greater than GHG emissions from light vehicles, but this big difference can be attributed to the average distance traveled by these types of vehicles. Owing to the lack of data the average travel distance employed to calculate the GHG emissions, belongs to Quebec and due to the great numbers of highways in Quebec this factor is bigger than the average travel distance by light vehicles. At the beginning of the period between 2002 and 2007, the trend was seen a growth, although this increase for light vehicles is not as significant as heavy vehicles. GHG emissions reached the peak by 2007 with 4,173.47 kt CO_2_-eq. There is a sudden reduction for both trends light and heavy vehicles but for light vehicles happened in 2009 and for heavy vehicles occurred in 2008, and then they are followed by a slight rise by 2016, reaching 2,889.64 kt CO_2_-eq by light vehicles, representing the highest GHG emissions over 13-year period and 4,023.8 kt CO_2_-eq by heavy vehicles.

### Emissions from fuel-based heating

**Fig. 10:**
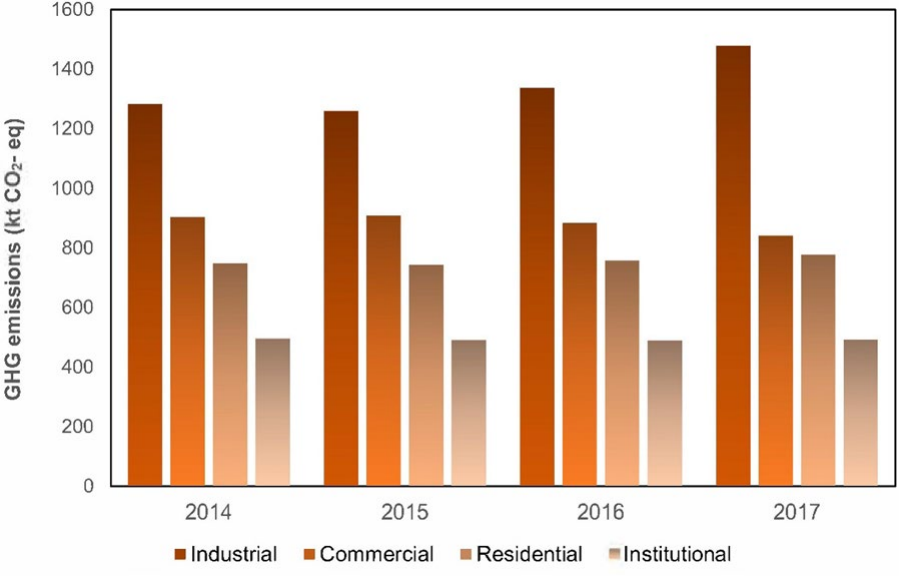
GHG emissions from natural gas-based heating during 2014 to 2017

Historically, Quebec has been a consumer of western Canadian natural gas. More recently, growing gas production in the U.S., a reversal of export points in Ontario, and additional interconnections between Ontario and Quebec have enabled higher rates of delivery of U.S. gas into Quebec. Énergir distributes gas to approximately 300 municipalities via over 10,000 km of pipelines. Enbridge Gazifère operates 932 km of pipelines and serves the Outaouais region. Énergir and Gazifère are provincially regulated by the Régie de l’énergie (Énergir 2019). GHG emissions from fuel-based heating are estimated in the present study by considering two sources, natural gas, and heating oil, while electricity has been studied in another sector which is only belonged to this area. The emission factors for natural gas based on different units and locations are provided in Additional file 1: Table S7. The emission factor which this study has considered for natural gas is 1.888 kg CO_2_-eq/m^3^ (Government of Canada, 2017b). This factor is chosen for two reasons: this emission factor was developed for Quebec which is the province of the case study of the present study, and its unit matches the activity data. The activity data of natural gas consumption was provided by Énergir. The reported natural gas consumption is categorized by four sectors, residential, commercial, industrial and institutional, as shown in Additional file 1: Table S8. Additional file 1: Figure S1 illustrates natural gas consumption in Montreal, where industry at 39% is shown to consume the highest rate of natural gas in Montreal, followed by commercial with 25%, while institutional and residential are the lowest and second-lowest consumers of natural gas in Montreal, with 22% and 14% respectively. Figure 10 shows the trend of GHG emission in Montreal by sector resulting from the use of natural gas during the period 2014 to 2017. Emissions by industry increased moderately by 2017, while GHG emissions by the commercial sector decreased slightly over the same period. GHG emissions by natural gas, meanwhile, experienced negligible growth by 2017, while Énergir reported that in 2017–2018 nearly 12.7 million m^3^ of natural gas was saved by customers in Montreal by energy-efficiency programs.

Oil is another source of heating in Montreal whose GHG emissions are estimated, employing a linear relation to multiply the total oil consumption by an emission factor. The Quebec government, it should be noted, is seeking to reduce the use of thermal coal and reduce by 40% the number of oil products used in the province. The Quebec government is also seeking to improve the efficiency of each source of energy by 15%. In order to meet these goals, Quebec will assist households and industries to reduce energy consumption. Among the energy-efficiency initiatives, building codes will be modified and energy-efficient renovations encouraged. The Quebec government will also encourage the use of Quebec-sourced energy, including hydro, wind, biomass, and geothermal. Households will receive credits for achieving self-sufficient energy production by wind and solar. Moreover, the Quebec government aims to increase the use of renewable energy by 25%, including 50% more biomass energy. A new hydrocarbon law is planned, and revenues generated from natural gas and oil production will be used to support further decarbonization (Flèche 2016). There is a lack of available data on oil consumption for heating in Montreal. CTV News (CTV Montreal 2019) reported on the Montreal mayor’s talks about heating oil in which she mentioned that the use of heating oil has decreased slightly over the years, but some 25,000 households still use it. Another 23,000 households use a dual-energy system. Since 23,000 households are using dual-energy, half of this number is considered and then added to the 25,000 households using oil. As such, it is considered that 36,500 households in Montreal are consuming oil for heating and, given that a typical home in a mild climate uses between 5000 kWh and 30,000 kWh of energy per year for heating (OVOenergy), the average is assumed, and this corresponds to 90 GJ. The oil consumed for meeting heating demand in Montreal is primarily gasoline, so the emission factor considered is 69.3 kg CO_2_-eq/GJ, meaning that the GHG emissions produced by heating oil is 227,650.5 t CO_2_-eq.

### Emissions from electricity-related activities

Hydroelectric power is the main source of energy used by Hydro-Quebec to produce electricity, which is why very little fossil fuel is used for generating electricity in Montreal compared with other jurisdictions (Hydro-Quebec 2017). Hydro is the source of 99% of the electricity in Montreal, provided by 63 hydroelectric generating stations, while the remaining 1% is generated by thermal power stations operating continuously to meet baseload energy needs (for instance, diesel generating stations), as well as some gas-fired facilities operating only when demand is high and hydroelectric facilities are working at maximum capacity (Hydro-Quebec 2017). Data pertaining to the amount of electricity used is obtained from annual reports published by Hydro-Quebec. Because Hydro-Quebec’s electricity is produced on an integrated network for the whole of Quebec, though, the data in these reports does not specify the electricity demand for Montreal in particular. For instance, the electricity consumed by customers comes from the overall network of Hydro-Quebec, not from a specific power plant or interconnection. Hydro-Quebec's transmission and distribution grid lines are interconnected and reach all customers over Quebec, with the exception of the off-grid generating stations (Hydro-Quebec 2019). Therefore, in order to estimate GHGs in Montreal, electricity consumption is assessed as a proportion of the Quebec population. The population of Quebec in 2016 was 8,164,361, while the population of Montreal proper was 1,765,616 (Statistics Canada 2016). According to Hydro-Quebec annual reports, electricity consumption in Quebec is categorized into three sectors: residential, large industries, and commercial including institutional and small industries. The large industry does not play a noticeable role in GHG emissions production in Montreal, and small industries are already considered in the commercial category. For these reasons, the large industry is not considered in this sector. Additional file 1: Table S9 is extracted from Hydro-Quebec annual report (Hydro-Quebec 2016) which represents the electricity consumption in Quebec and electricity consumption by Montreal in 2016. To calculate the amount of GHG emissions produced by electricity consumption in Montreal, the above amount is multiplied by the population of Montreal and then divided by the population of Quebec. Additional file 1: Table S10 clarifies the electricity consumption in Montreal from 2006 to 2016 to establish a trend of GHG emissions by electricity consumption over the period under study.

The emission factors collected from different sources are shown in Additional file 1: Tables S11-13. The calculation is carried out based on emission factors and ratio of electricity generation. Additional file 1: Table S12 shows the emission factors for the operation of each source, while gives the emission factors for LCA of the sources (Vuyk 2005). Since the present study is evaluating municipal GHG emissions for a single year (2016), only the emission factors for operation are considered in the calculation. It should also be noted that the emission factors are collected from different sources with different units. As shown in Additional file 1: Table S12, the emission factor for operation in electricity generated by hydropower is zero. As mentioned above, in Quebec 99% of electricity is generated by hydropower and just 1% is generated by diesel- and gasoline-fired thermal power plants. The emission factor for electricity generated by thermal power is considered the middle number of oil-fired plants. Therefore, the total GHG production in Montreal in 2016 by electricity is calculated 202.014 kt CO_2_-eq.

**Fig. 11:**
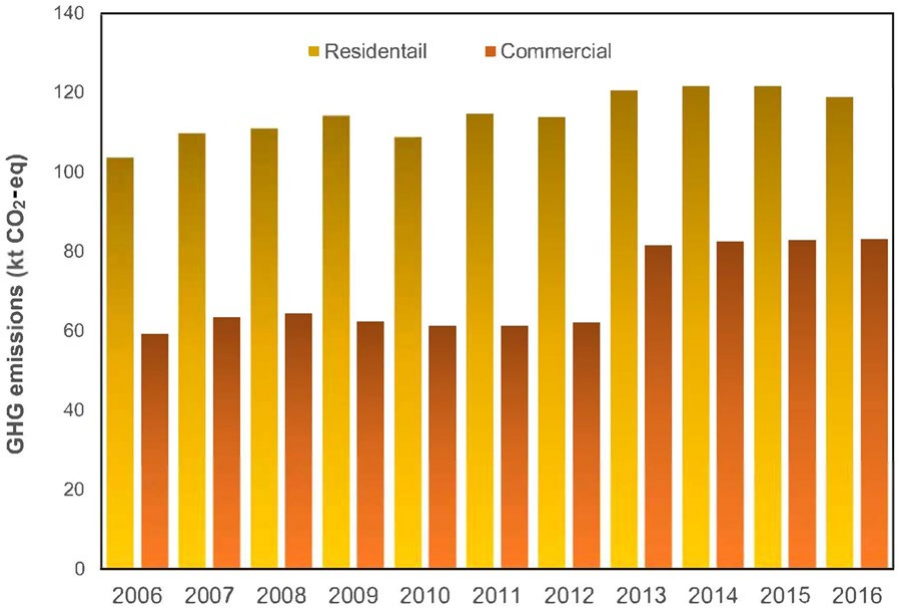
GHG emissions from thermal power-based electricity production

Figure 11 shows the GHG emissions due to electricity generation by thermal power for both residential and commercial use. The GHG emissions produced by commercial is, overall, less than GHG emissions by residential towing to the more usage by this category. It stood at 60 kt CO_2_-eq in 2006, although GHG emissions produced in the residential sector is approximately 100 kt CO_2_-eq. GHG emissions by commercial are found to have increased slightly by 2008, even though a reduction of about 4 kt CO_2_-eq is seen by 2011, and the trend holding in 2012. Sudden growth is then seen in 2013, peaking at 80 kt CO_2_-eq and then remaining stable for the remainder of the period under study. This category is found to have emitted 59.28 kt CO_2_-eq in 2016. The GHG emissions produced by the residential user category have seen a more stable trend compared to commercial, rising slightly by 2009 to approximately 120 kt CO_2_-eq, then decreasing by 2010 to 105 kt CO_2_-eq. The trend is found to have risen to 110 kt CO_2_-eq by 2011, remaining at the same level in 2012. Peaking in 2013 at 125 kt CO_2_-eq, GHG emissions in this category had decreased slightly by the end of the period under study. The electricity consumed by the residential category is found to have been associated with 103.65 kt CO_2_-eq emissions from thermal power generation. The calculation carried out, it should be noted, is based on three factors: total electricity consumption, electricity generation ratio, and emission factor. The emission factor and the generation ratio (0.01 for thermal power) are constant values in this method and only the electricity consumption varies. Moreover, the GHG emissions produced by electricity generated from hydropower is zero, given that the corresponding emission factor is zero.

### Emissions from solid waste disposal

In recent years, Montreal has experienced a considerable increase in recovered materials, except for organic matter. In 2008, the rate of recovery of recyclables was 53%. The rate was 54% for hazardous household waste and 43% for construction, renovation, and demolition (CRD) waste and bulky refuse. However, for organic matter, the recovery rate was only 8%, while the overall recovery rate for Montreal was 31%. Anaerobic decomposition of MSW in landfills generates about 60% CH_4_ and 40% CO_2_, together with other trace gases (Jha et al. 2008). Residential waste includes organics, leaf, and yard, municipal hazardous or special waste, other recyclable materials such as wood, metal, and tires, as well as construction and demolition materials. Data about the amount of waste collected in Montreal is obtained from the Government of Quebec and is provided in three categories: household waste, industrial, commercial, and institutional waste, and CRD waste. The amount of municipal solid waste for Montreal is shown in Additional file 1: Table S14. It should be noted that, for the estimation of GHG emissions, CRD waste is not included.

**Fig. 12:**
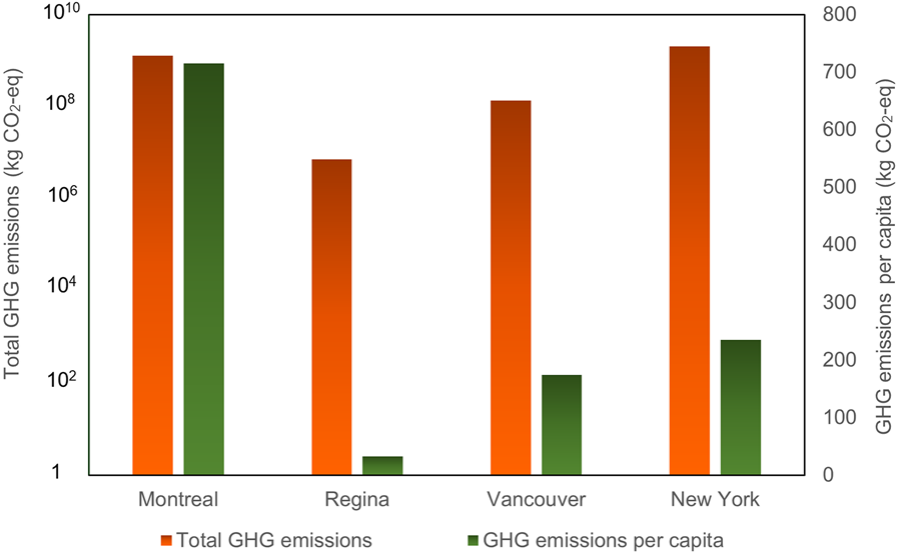
GHG emissions from solid waste disposal in different cities in 2016

The formula is followed by CH_4_ correction factor which, based on other studies, is assumed to be 0.6, while the degradable organic carbon in waste is assumed to be 0.15 kg of carbon per kg of waste. The fraction of DOC dissimilated, meanwhile, is 0.77, while the fraction of CH_4_ in landfill gas is assumed to be 0.5. The default amount for recovered methane is 0 due to the lack of CH_4_ recovery. Another factor considered is the conversion of C to CH_4_, which is 16/12. According to some studies (Gurjar et al. 2004), the oxidation factor has been considered to be zero. The methane generation which is calculated by the developed methodology should be multiplied by the global warming potential for methane is 25. So, the estimation shows that 1,262.38 kt CO_2_-eq was produced by this sector in 2016 in Montreal. Figure 12 shows the comparison of GHG emissions from solid waste disposal in Montreal, New York City, Vancouver, and Regina. In comparison to other cities, the amount of GHG emissions produced in Montreal is higher than that of Regina, which produced 7056 t CO_2_-eq in 2016, although it is less than New York City (with a population of 8.615 million in 2016, having 2,021,979 t CO_2_-eq, i.e., the highest emission among these cities), it also should be mentioned that the scale of these two cities is different, with Montreal being much more populous than Regina and more comparable in scale to cities such as Vancouver and New York City. In a comparison of the same cities on a per capita basis, the total GHG emissions produced in Regina in 2016 are less than that in other cities.

### Emissions from wastewater treatment

The Montreal region alone produces two-thirds of the wastewater in Quebec. The treated wastewater in Montreal, obtained from Ville de Montreal, is 829,396, 800.0 m^3^. Following the methodology, the amount of treated wastewater is multiplied by the concentration of BOD and nitrogen, which are 35 (*g*/m^3^) and 60 (*g*/m^3^), respectively (Henze and Comeau 2008). Additional file 1: Tables S15 and S16 the emission factors for CO_2_, CH_4_ and N_2_O, which are 0.15 kg CH_4_ per kg BOD and 0.0005 kg N_2_O-N per kg N, respectively. The GWP values are shown in Additional file 1: Table S17.

**Fig. 13:**
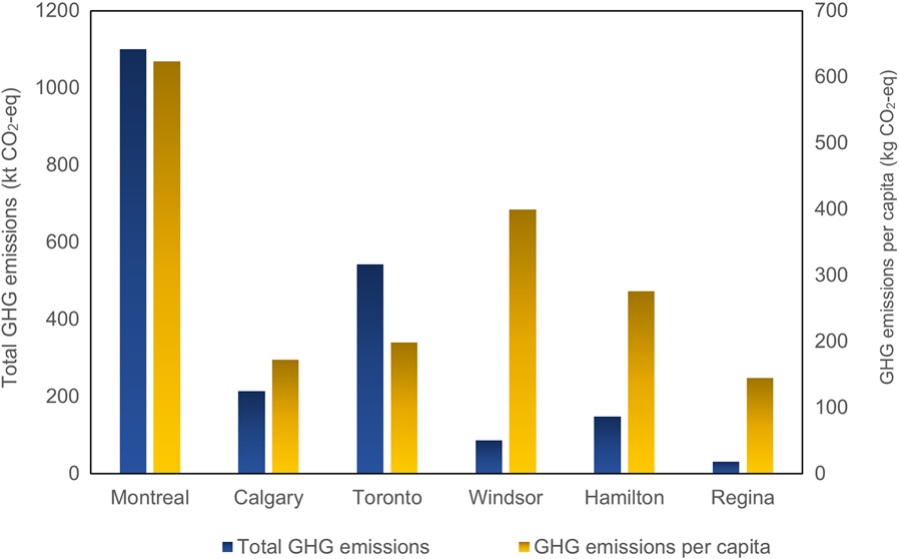
GHG emissions by wastewater in 2016

The treated wastewater for cities, Toronto, Calgary, Hamilton, Windsor and Regina has been extracted from MBN Canada report (Municipal Benchmarking Network Canada, 2016) shown in Additional file 1: Figure S2 and calculated based on the developed methodology to obtain GHG emissions. The amount of wastewater produced in Montreal is the highest among the cities being compared, Calgary, Toronto, Windsor, Hamilton and Regina. According to the comparison of treated wastewater (Additional file 1: Figure S2), Montrealers are the highest producers of wastewater, and this city is one of the major treaters of wastewater. Then, it is followed by Toronto with small difference and Calgary and Windsor stand as the third and fourth highest treated wastewater. Hamilton and Regina with bigger difference and different scale too have the least and second-least treated wastewater in both comparison of total treated wastewater and wastewater per capita. Wastewater produced 1,100.22 kt CO_2_-eq in Montreal in 2016 which is compared with GHG emissions from wastewater treatment in Calgary, Toronto, Windsor, Hamilton and New York in Fig. 13. Comparing GHG emissions from wastewater from Montreal with the cities, it is found Montreal is the largest driver of GHGs by wastewater. However, GHG emissions from wastewater treated from Toronto in 2016 accounts for 458.19 kt CO_2_-eq, standing as the second-largest driver of GHG emissions by wastewater despite its population that was almost two times greater than the population of Montreal. Moreover, Montreal’s treated wastewater amount is up to two times greater than the amount of wastewater treated in Toronto. Although treated wastewater per capita is approximately the same in Toronto, Calgary, and Windsor, Toronto is found to have emitted the highest total GHG emissions among these three cities. Even though New York City is more populous and bigger city in comparison to Windsor and Calgary, the GHG emissions produced by wastewater in New York City is lower than these two cities. Regina accounts for the lowest emissions in this sector. Residents in Hamilton and Regina producing 153.30 kg CO_2_-eq and 32.1 kg CO_2_-eq as the total GHG emissions from wastewater in 2016, produced GHG emissions less than 1 kg CO_2_-eq per capita.

### Carbon sequestration from greenspace

To account for the carbon offset due to the absorption of CO_2_ by trees in urban parkland, the number of trees is multiplied by the absorption factor per tree. There is no data for the total number of trees in Montreal island, but the report of MBN Canada (Municipal Benchmarking Network Canada, 2016) has published information in terms of two categories: maintained parkland and natural parkland. Hectares of maintained and natural parkland per 100,000 people have been noted to be 124 and 106, respectively Montreal, as of 2016. Based on the average amount of space occupied by a single tree multiplied by the total area of parkland in Montreal, the number of trees is assumed to be 481,667. The absorption factor, it should be noted, is defined as the average amount of CO_2_-eq which can be absorbed by a single tree per year, which is 22 kg CO_2_-eq (Ministry of Environment British Columbia 2016). The carbon offset from trees in Montreal in 2016 is thus calculated to be 10,596 t CO_2_-eq. To find the net GHG emission produced in Montreal in 2016, the carbon offset amount due to the absorption of CO_2_ by trees in urban parkland is subtracted from the total emissions to obtain the net GHG emissions emitted to the atmosphere from Montreal in 2016.

### Total GHG emissions from Montreal

**Fig. 14:**
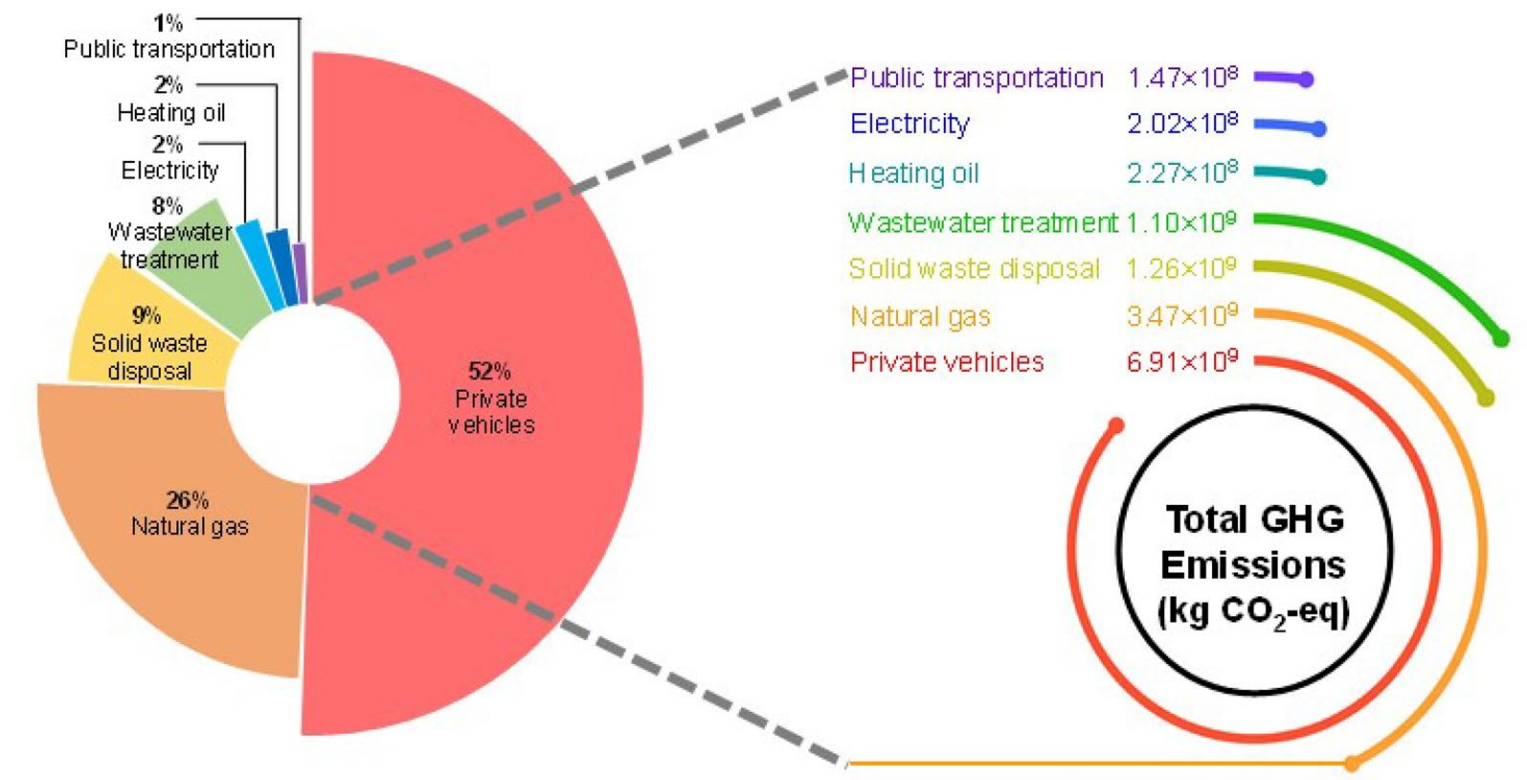
The ratios of total GHG emissions from Montreal in 2016

A total of 13.310 Mt CO_2_-eq is found to have been emitted in Montreal in 2016 with consideration of CO_2_ absorption by green space; without emission absorption, the total GHG emissions in Montreal in 2016 would be 13.32 Mt CO_2_-eq. The results obtained are applied to different sectors considered in the case study (see Fig. 14). Private vehicles accounted for 52% of total GHG emissions in Montreal in 2016, standing as the largest driver of GHG emissions. The action plan has been taken to reduce GHG emissions by private vehicles. The city has also invested public transportation to improve its services and encourage resident to use this type of transit. Natural gas constituted by 26% as the second-largest driver of GHG emission. The city is working to reduce the use of fuel and increase the use of renewable energy for heating. The financial incentives are available for buildings that are moving to renewable energy, instead of natural gas. Solid waste disposal with 1,262.38 kt CO_2_-eq, representing the third-largest GHG emissions in Montreal in 2016. Wastewater treatment, due to the large amount of treated wastewater, is found to have been the fourth-highest level of GHG emissions, accounting for 8% of total GHGs. Although only 1% of electricity is generated by thermal power, 2% of the total GHG emissions in Montreal in 2016 is attributed to electricity. Whereas oil used by a few households in the city for heating, accounted for 2% of the total GHG emissions with 227.65 kt CO_2_-eq. Urban transportation network with primary fuel of diesel constituted only 1% of total GHG emissions in Montreal in 2016.

**Fig. 15:**
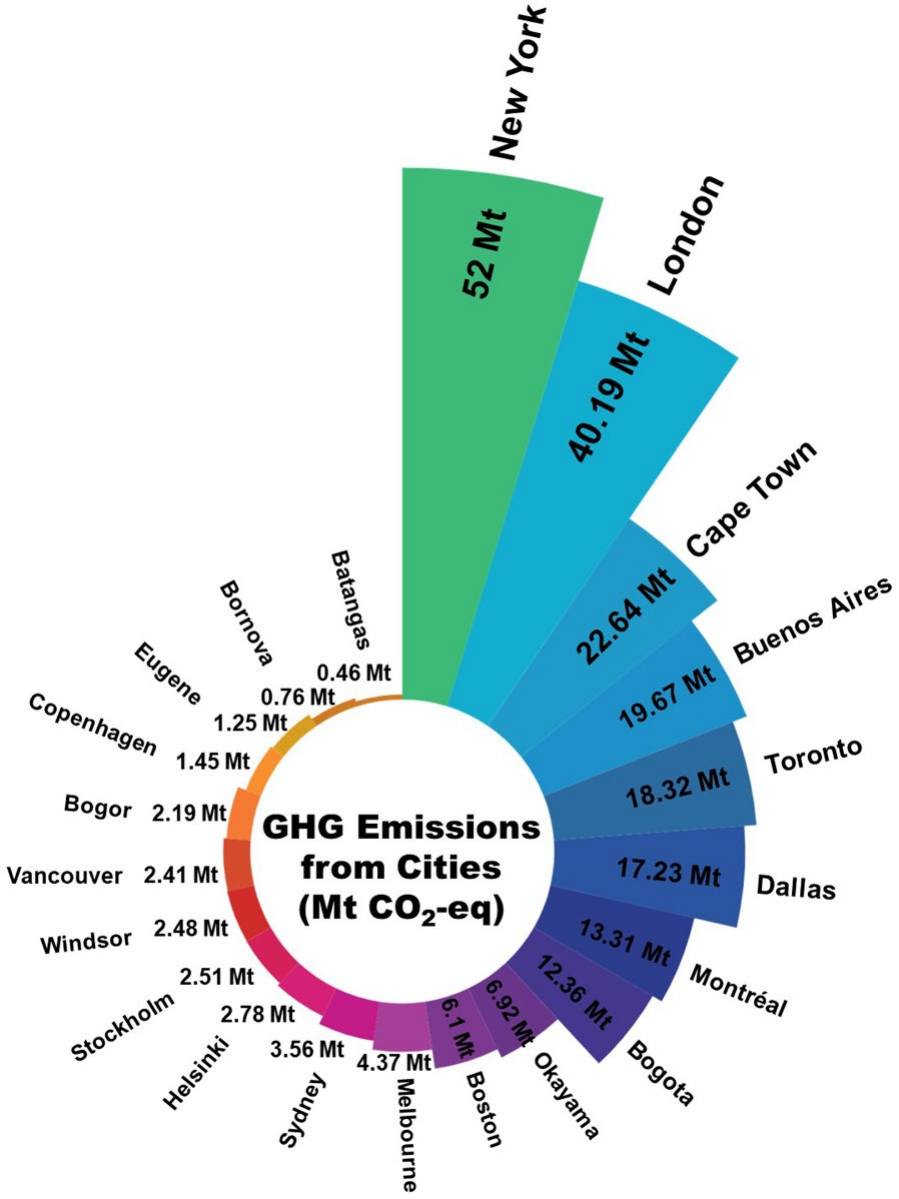
GHG emissions from other cities in 2016

A GHG emission inventory of New York City (City of New York, 2017) is one of the reports compared with the results for Montreal. This report categorizes the factors contributing to municipal GHG emissions as follows: (i) energy used by buildings and other stationary sources, and fugitive emissions from natural gas distribution within the city limits of NYC, (ii) on-road transportation, railways, marine navigation, and aviation within city limits, (iii) wastewater treatment within the city boundary and solid waste generated within the city but disposed outside of the city. The total GHG emissions produced in New York City in 2016 was 52 Mt CO_2_-eq with around 8.615 million population, and each resident in New York City was responsible for 6.1 MT CO_2_-eq in 2016 (City of New York 2017). This report shows that the stationary energy sector, including the combustion of natural gas (31%), the use of electricity (25%), and the combustion of gasoline (24%), produced the highest GHG emissions in New York City in 2016 among the categories considered. The second-highest emitter was transportation, while the third was waste and wastewater. When comparing Montreal and New York City, it is noted that the highest-emitting sector in Montreal is private transportation. While the data on emissions from the transportation sector in New York City is not further categorized into private and public, even with private and public transportation combined it is only the second-largest driver of GHG emissions. Waste and heating, meanwhile, are found to be at the same level in both cities. In comparing Montreal to Toronto for the same year, the transportation sector accounts for over 40% of Toronto’s overall GHG emissions, approximately the same as the proportion in Montreal. The results obtained are also compared with other cities such as Helsinki in Finland, Batangas in the Philippines, Okayama in Japan, Dallas in the United States, and others, as shown in Fig. 15, which illustrates that among all these cities the highest GHG emission was produced by New York City in 2016 while the second-highest producer was, United Kingdom. Cape Town, South Africa, Buenos Aires, Argentina, Dallas, in the United States, and Toronto ranked third, fourth, fifth, and sixth as GHG emitters, with Montreal following Toronto in the seventh position in this comparison.

### Sensitivity analysis

A sensitivity analysis is further conducted to determine which factor has the most significant effect on total GHG emission production and assess the results (Sun et al. 2014). Due to huge number of combinations, the evaluations has been conducted by fractional factorial analysis. The methodology developed in the present study includes many factors, such as emission factors, fuel economies, travel distances, average weight of trains and concentration of BOD and nitrogen. To clarify the results, the factors in this study are divided into two categories: emission factors and other factors. The sensitivity analysis is carried out using Minitab software.

There are 10 emission factors evaluated, the emission factor of heating fuel, the emission factor of energy (gasoline), the emission factor of energy (diesel), the emission factor of energy (biodiesel), the emission factor of suburban public transportation type, the emission factor of fuel consumption (light vehicles), emission factor of fuel consumption (medium and heavy vehicles), the emission factor for CH_4_ in wastewater, the emission factor for N_2_O in wastewater, and the emission factor for electricity generated by oil-fired power plants, as shown in Additional file 1: Table S18. Additional file 1: Table S19 demonstrates the alias relationships for 2^10–4^ fractional factorial analysis and solutions of 2^10–4^ fractional factorial analysis, respectively.

**Fig. 16:**
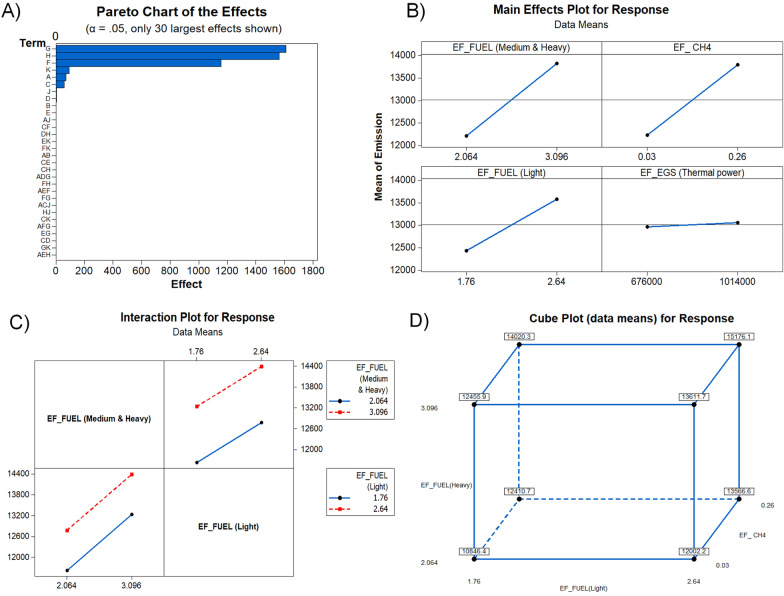
Factorial analysis results: **A** Pareto chart of the effects **B** Main effect plots for different factors, **C** Interaction plot for response, **D** cubic plot for response

Figure 16A indicates that factors G, H, F, K, A, C, J, D had significant effects on the total GHG emissions in Montreal. Since private vehicles are found to have produced the highest rate of GHG emissions in Montreal in 2016 at 6.91 Mt CO_2_-eq, “G”, standing for emission factor of fuel consumption (medium and heavy vehicles) plays the primary role in this assessment. The GHG emitted to the atmosphere only by heavy vehicles in Montreal in 2016 was 4.02 Mt CO_2_-eq. “H”, meanwhile, which represents the emission factor for CH_4_ in wastewater treatment, is the second-most significant factor and is associated with 1.1 Mt CO_2_-eq of emissions, accounting for 8% of the total. The third-most significant factor, represented as “F”, is the emission factor for light vehicles, where this sector produced 2.88 Mt CO_2_-eq in emissions. The emission factor for electricity generated by oil-fired power plants (represented as “K”) is the fourth-most important factor, although electricity is found to have been one of the least significant drivers of GHG emissions in Montreal in 2016. The next factor, “A”, is the emission factor for heating, where natural gas is the next-most significant factor after private vehicles. Figure 16B illustrates the main effects plot which shows the variation of the four most important emission factors recognized in pareto chart. The emission factor for heavy vehicles (G) plays the main role among these emissions factors, followed by the emission factors for methane in wastewater, light vehicles, and thermal power. Figure 16C shows the interactions between two factors, while Fig. 16D shows the relationships of emission factors for heavy vehicles, light vehicle and methane in wastewater treatment.

In addition, the significance of the following factors is also evaluated: TTD (total travel distance of suburban public), AW (average weight of suburban public), ATD_VEH (average travel distance by light and heavy vehicles), DOC_SW (Degradable organic carbon in solid waste), F_DOC (fraction of DOC dissimilated), CBOD (concentration of BOD in raw wastewater), CN (concentration of nitrogen in raw wastewater) and Ratio_ELEC (electricity generation ratio), and oil consumption for heating. In total there are 10 factors. Additional file 1: Table S20 shows the terms and factors evaluated in this section, while Additional file 1: Table S21 illustrates the relations for fractional factorial analysis.

**Fig. 17:**
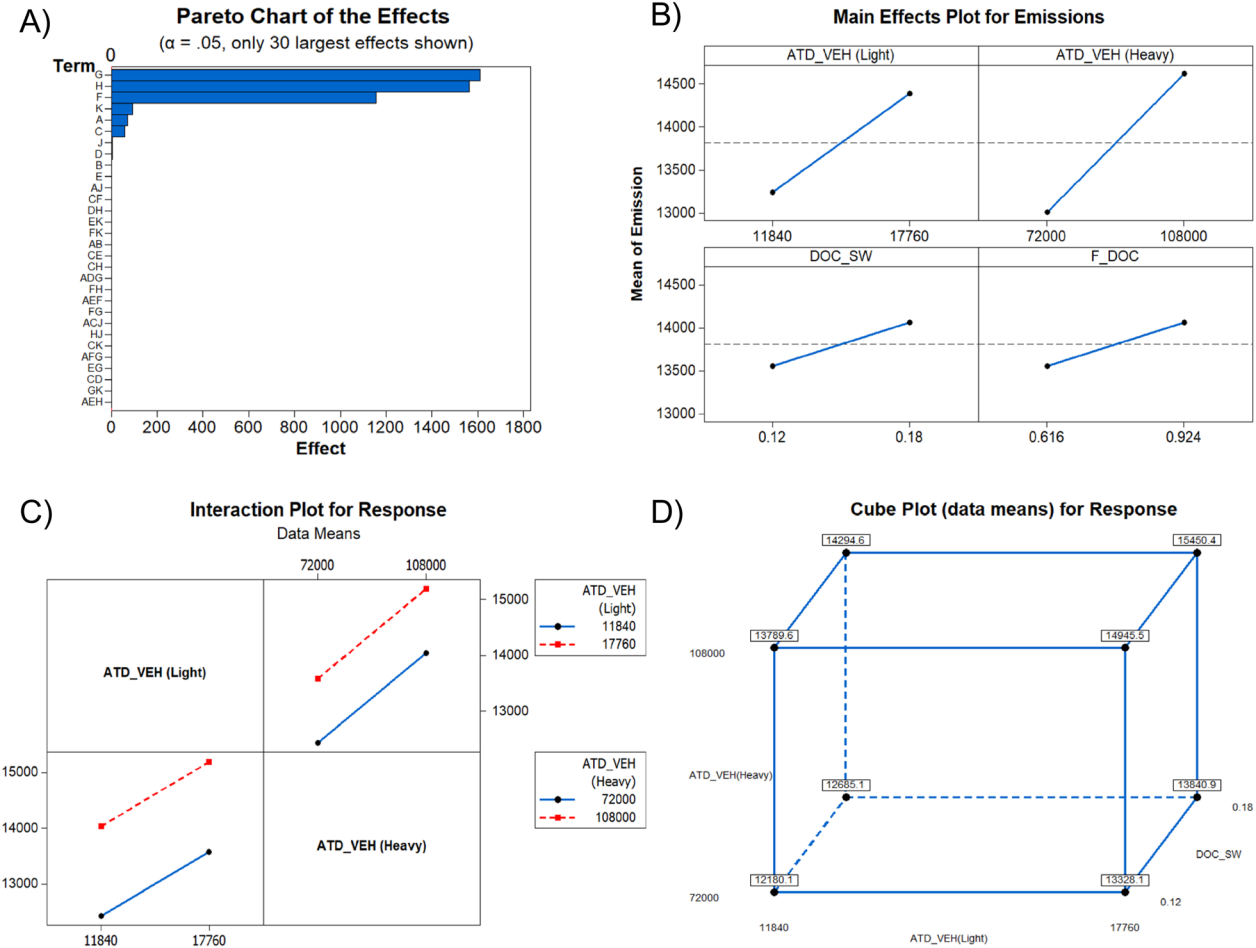
Factorial analysis results: **A** Pareto chart of the effects **B** Main effect plots for different factors, **C** Interaction plot, **D** cubic plot

Figure 17A shows that term “D”, which represents average travel distance for heavy vehicles, has the most important impact among the factors, while average travel distance for light vehicles stands as the second-most important factor “C”, and was the largest driver of GHG emissions with natural GHG in Montreal in 2016. “E”, representing degradable organic carbon, and “F”, standing for fraction of degradable organic carbon dissimilated, are the third- and fourth-most significant factors, respectively, since municipal solid waste disposal constituted only 9% of the total GHG emission produced in Montreal in 2016 with 605.94 kt CO_2_-eq. Oil consumption (“K”) is found to have played a minimal role as the fifth factor, while the concentration of BOD_5_ is the sixth-most significant factor, whereas wastewater is found to have produced the second-highest GHG emissions in Montreal in 2016. In other cities, stationary energy, including electricity and heating, is the primary driver of GHGs, such that the electricity generation ratio is likely to play the primary role in determining the emissions derived from electricity generation. However, in the case of Montreal, where 99% of electricity is from hydropower and only 1% is from oil-fired power plants, electricity generation is just the seventh-largest driver of GHG emissions. Figure 17B further shows the impact of the variation of these factors on the total GHG emissions. Figure 17C shows the interaction of two factors and Fig. 17D shows the cubic plot for the response impacted by the average travel distance of heavy vehicles, average travel distance of light vehicles, and degradable organic carbon in municipal solid waste disposal.

## Conclusions

In the present study, a detailed methodology for the assessment of urban GHG emissions from human activities such as heating and electricity demand, transportation, and waste processing and wastewater processing was developed to assess urban GHG emissions. The methodology is then applied to Montreal as a case study and it can also be applied for the assessment of other cities. The data has been obtained from different organizations, annual reports, academic publications, and websites. A wide range of emission factors has been considered with different units and different sources in each sector for different locations. A comprehensive assessment of GHG emissions in Montreal was conducted based on the developed method. Montreal is committed to reduce GHG emissions by 30% compared to 1990 levels by 2020 and by 80% by 2050 (City of Montreal 2021). The city identified four sustainable development priorities: (i) reduce GHG emissions and dependence on fossil fuels, (ii) add vegetation, increase biodiversity and ensure the continuity of resources, (iii) ensure access to sustainable, human-scale and healthy neighborhoods, (iv) make the transition toward a green, circular and responsible economy (Sustainable Development Montreal, 2016). Action plans have been developed under these priorities to meet the targets. The assessment of urban GHG emission can help the city to identify the key factors contributing to GHG emissions, evaluate the efficacy of mitigation measures, and achieve carbon–neutral objective.

### Supplementary Information

**Additional file 1. **Supplementary tables and figures.

## Data Availability

The authors declare that the data supporting the findings of this study are available within the paper and its Supplementary Material.

## Abbreviations

BOD: Biochemical Oxygen Demand
COD: Chemical Oxygen Demand
CRD: Construction, Renovation, and Demolition
GHGs: Greenhouse Gases
LCA: Life Cycle Assessment
MBN: Municipal Benchmarking Network
MSW: Municipal Solid Waste
RTM: Régie des Transports Métropolitains
SWD: Solid Waste Disposal
WWTPs: Wastewater Treatment Plants
EP: Per Person
