# Supplementary material for: Assessment of urban greenhouse gas emissions towards reduction planning and low-carbon city: a case study of Montreal, Canada
Source: Environ Syst Res (Heidelb). 2024 Apr 16;13(1):12. doi: 10.1186/s40068-024-00341-y (PMC11021249; doi:10.1186/s40068-024-00341-y)
Supplement: Supplementary file 1 — Additional file 1. Supplementary tables and figures. [file 40068_2024_341_MOESM1_ESM.pdf]

SUPPLEMENTARY MATERIAL

Assessment of Urban Greenhouse Gas Emissions towards Reduction Planning and Low-Carbon City: A Case Study of Montreal, Canada

*Shadnoush Pashaei and Chunjiang An**

Department of Building, Civil and Environmental Engineering, Concordia University, Montreal, H3G 1M8, Canada

Table S1. Energy consumption by STM in 2016 (Societe de Transport de Montréal STM, 2016)

Indicator	Amount	Unit
Service offering	163,265	thousands of km traveled
Metro	79,299	thousands of km traveled
Surface network	83,965	thousands of km traveled
Total energy consumption	3,873.1	PJ (10^{15} joules)
Total energy consumption from non-renewable sources	2,378.7	PJ (10^{15} joules)
Total energy consumption from renewable sources	1,494.4	PJ (10^{15} joules)
Diesel	1,872	TJ
Gasoline	26	TJ
Electricity	1,434	TJ
Biodiesel	60	TJ
Total energy consumption per passenger-km	1,110	kJ
Total energy consumption per passenger-km	1,110	kJ
Total energy consumption per seat-km	307	kJ

Table S2. Emission factors for fuel used in public transportation.

Fuel type	Amount (kg CO_2-eq /unit)	Unit	Location	Reference
**Gasoline**	69.30	GJ	Portugal	(Agência Portuguesa do Ambiente, 2017)
	3,200	Mg	China	(Zhang et al., 2007)
**Diesel**	74.07	GJ	Portugal	(Agência Portuguesa do Ambiente, 2017)
	2.6712	Liter	Australia	(Australian Transport Assessment and Planning (ATAP), 2016)
	10.21	gallon		(U.S. EPA, 2018)
**Biodiesel**	71.42	GJ	Portugal	(Agência Portuguesa do Ambiente, 2017)
	9.45	gallon		(U.S. EPA, 2018)
**Electricity**	0.002-0.048	KWh		(Zhang et al., 2007)

Table S3. Energy consumption by STM from 2006 to 2015 (Societe de Transport de Montreal STM, 2016)

Energy consumption (TJ)	Diesel	Gasoline	Electricity	Biodiesel
2006	1,787	23	1,042	-
2007	1,697	31	1,237	1
2008	1,705	31	1,355	51
2009	1,822	30	1,343	65
2010	1,927	15	1,347	66
2011	1,997	24	1,368	67
2012	1,981	28	1,380	69
2013	1,974	24	1,394	66
2014	1,898	27	1,431	69
2015	1,856	25	1,442	76

Table S4. Number of vehicles registered from 2002 to 2016 (Societe de Anssurance Automobile Québec, 2007; 2008; 2009; 2016)

	2002	2003	2004	2005	2006	2007	2008	2009	2011	2012	2013	2014	2015	2016
**Personal purpose**														
**Automobile and light truck**	634323	648366	653669	655931	7E+05	668059	7E+05	702231	716842	720948	730061	7E+05	747565	760063
**Motorcycle**	1421	1708	1988	2289	2824	3336	13020	12568	12327	13668	14322	15079	15710	16211
**Moped**	0						4173	4496	4935	5291	5402	5354	5244	5273
**Motor home**	0						668	732	692	663	653	626	634	624
**UIPCP**														
**Automobile or Light truck**	120307	125656	128113	131025	1E+05	132275	1E+05	11865	100963	101020	102793	1E+05	105051	106139
**Taxi**	3541	3527	3489	3470	3399	3347	3313	3289	3306	3265	3260	3273	3234	3212
**Bus**	2309	2279	2262	2291	2290	2296	2370	2317	2709	2307	2378	2303	2286	2362
**School bus**	620	655	722	859	861	886	903	733	933	856	1028	1033	1054	1049
**Truck or tractor unit**	21688	22744	23817	24841	24779	24906	24092	23283	23385	23896	24319	24545	25055	25333
**Tool vehicle**	7905	8152	8446	8819	8967	9052	5416	5610	5788	5959	6140	6132	6166	6298
**Motorcycle, moped,motor home and others**	0	0					3526	3566	3719	3887	3906	3818	3949	4098
**Restricted circulation**	0	0					1119	1502	2063	1017	946	865	806	827
**Off network - Snowmobile**	4005	4514	4180	4076	3843	3788	3781	3736	3294	3161	3222	3153	3022	2899

	2002	2003	2004	2005	2006	2007	2008	2009	2011	2012	2013	2014	2015	2016
**Off-grid - all-terrain vehicle**	7615	7991	8059	8338	8542	9032	9323	9414	9329	9017	9077	9050	9032	8908
**Off-grid - tool vehicle**	718	776	815	760	858	998	6450	6751	7769	8019	8055	7854	7951	7876
**Off-grid - automobile, light truck, moped, bus, truck or tractor unit and others**	6260	6257	6382	6659	6878	7209	1137	1326	1167	1229	1104	1269	1320	1356

These categories are defined in the SAAQ reports as follows:

Personal purpose means that the authorization to circulate was obtained by an individual or more of co-owners, and the use of the vehicle is mainly for personal purposes. **Utilization institutional, professional or commercial purpose** means that the authorization to circulate was obtained by a legal person, a government, a public organization, a society, a company, an agricultural producer, or a professional working on their own. personal purpose is defined by who has not Canadian citizenship and who is a staff member director of the organization of international civil aviation or representative of a member state of this organism, or who is consular officer, a delegate from a foreign country or his assistant. **Off-network** means a vehicle using outside the ordinary road network, either on private land or in a locality not connected to the road network.

This study categorizes vehicles according to their weight: light, medium, or heavy.

- light vehicles weigh less than 4,500 kg (e.g., cars, vans, or light pickups)
- Medium vehicles weigh between 4,500 kg and 9,000 kg (e.g., heavy-duty pickups and medium-size pickups)
- Heavy vehicles weigh more than 9,000 kg (e.g., garbage trucks and tandem dump trucks)

Table S5. Number of registered vehicles based on this study categorized

Types of vehicles	2002	2003	2004	2005	2006	2007	2008	2009	2011	2012	2013	2014	2015	2016
**Light Vehicles**	759592	779257	787259	792715	798351	8E+05	831130	7E+05	844847	849759	861343	869938	882193	896447
**Heavy vehicles**	51120	53368	54683	56643	57018	58167	53472	53170	54374	54444	55323	55339	55886	56081

Table S6. Road transportation emission factors

Vehicle	Fuel	Emission factor (kg CO_2-eq /unit)	Unit	Location	Reference
Light vehicle	Gasoline	2.20	Liter	BC, Canada	(Ministry of Environment British Columbia, 2016)
	Diesel	74.07		Portugal	(Agência Portuguesa do Ambiente, 2017)
		2.58	Liter	BC, Canada	(Ministry of Environment British Columbia, 2016)
		2.671	Liter	Australia	(Australian Transport Assessment and Planning (ATAP), 2016)
	Natural gas	2.73	kg	BC, Canada	
		1.561	Liter	Australia	(Australian Transport Assessment and Planning (ATAP), 2016)
	Electricity	0.010	kWh	BC, Canada	(Ministry of Environment British Columbia, 2016)
Medium Vehicle	Gasoline	2.20	Liter	BC, Canada	(Ministry of Environment British Columbia, 2016)
	Diesel	2.58	Liter	BC, Canada	(Ministry of Environment British Columbia, 2016)
		2.671	Liter	Australia	(Australian Transport Assessment and Planning (ATAP), 2016)
	Natural gas	2.73	kg	BC, Canada	(Ministry of Environment British Columbia, 2016)
Heavy vehicle	Gasoline	2.20	Liter	BC, Canada	(Ministry of Environment British Columbia, 2016)
	Diesel	74.07		Portugal	(Agência Portuguesa do Ambiente, 2017)
		2.58	Liter	BC, Canada	(Ministry of Environment British Columbia, 2016)
		2.671	Liter	Australia	(Australian Transport Assessment and Planning (ATAP), 2016)
	Natural gas	2.73	kg	BC, Canada	(Ministry of Environment British Columbia, 2016)

Table S7. Emission factors for natural gas

Location	Emission factor (kg CO_2-eq per unit)	Unit	Reference
	53.6	Scf	(U.S. EPA, 2018)
Canada	56	GJ	(Government of Canada, 2017)
	56.11	GJ	(Government of Canada, 2017)
The Netherlands	63.1	GJ	(SenterNovem, 2005)
British Columbia, Canada	49.58	GJ	(Ministry of Environment British Columbia, 2016)
Quebec	1.887	M^3	(Government of Canada, 2017)
Ontario	1.888	M^3	(Government of Canada, 2017)
British Columbia	1.926	M^3	(Ministry of Environment British Columbia, 2016)
	0.0544	Scf	(U.S. EPA, 2018)
New York	50.411	GJ	(The City of New York, 2017)

Table S8. Natural gas consumption in the Montreal Island (Energir, 2019)

**Sector**	**2013-2014 (M^3)**	**2014-2015 (M^3)**	**2015-2016 (M^3)**	**2016-2017 (M^3)**	**2017-2018 (M^3)**
Commercial	477.3	482.8	475.5	445.9	446.7
Industrial	674.5	659.6	690.1	762.4	848.6
Institutional	263.7	260.7	258.6	263.2	255.3
Residential	397.2	392.2	398.1	413.2	407.6
Total	1,812.6	1,795.3	1,822.3	1,884.8	1,958.2

Table S9. Total electricity consumption in Quebec in 2016 by categories (Hydro-Quebec, 2016)

**Segment**	**Electricity consumption in Quebec (GWh)**	**Electricity consumption in Montreal (GWh)**
Residential	65,065	14,070.88
Commercial, institutional and small industrial	45,483	9,836.10

Table S10. Total electricity consumed in Montreal (Hydro-Quebec, 2012; 2014)

**Year**	**Sector**	**Total Electricity Consumption in Quebec (GWh)**
2016	Residential	14,070.88
	Commercial	98,36.10
2015	Residential	14,393.76
	Commercial	9,804.10
2014	Residential	14,721.61
	Commercial	9,772.52
2013	Residential	14,269.41
	Commercial	9,649.47
2012	Residential	13,479.20
	Commercial	7,349.13
2011	Residential	13,569.82
	Commercial	7,259.60
2010	Residential	12,874.76
	Commercial	7,323.61
2009	Residential	13,512.72
	Commercial	7,385.46
2008	Residential	13,137.08
	Commercial	7,618.37
2007	Residential	12,985.48
	Commercial	7,515.21
2006	Residential	12,266.64
	Commercial	7,015.44

Table S11. Emissions factors for electricity consumption by place (Koffi et al., 2017)

Location	Emission factor (kg CO_2-eq /GJ)
Austria	86.11
Belgium	111.66
Germany	196.11
Denmark	211.11
Spain	177.5
Finland	116.11
France	40.55
United Kingdom	182.77
Greece	324.16
Ireland	241.66
Italy	196.66
Netherlands	198.88
Portugal	208.33
Sweden	21.94
Bulgaria	251.66
Cyprus	283.05
Czech Republic	222.77
Estonia	442.5
Hungary	188.33
Lithuania	48.33
Latvia	156.38
Poland	329.16
Romania	301.11
Slovenia	167.22
Slovakia	98.05
China	0.23-0.28
Russia	175.55

Table S12. Emission factors for electricity operation

Fuel type	Emission factor for operation (kg CO_2-eq /unit)	Unit	Location	Reference
Hydroelectric	0.376	m^3	Japan	(Shimizu, Y and others, 2012)
	0	MWh		(Koffi et al., 2017)

Table S13. Emission factor for electricity (Life cycle assessment)

Fuel type	LCA Emission factor (kg CO_2-eq /unit)	Unit	Location	Reference
**Hydroelectric**	0.002-0.048	KWh		(Zhang et al., 2007)
	0.015	KWh	India	(Prakash and Bhat, 2012)
**Oil-fired power plant**	790-900	MWh		(William Steinhurst and Schultz, 2012)

Table S14. Solid waste disposal in Montreal by region (Environment Quebec, 2018)

Region	Household waste (tonne)	Industrial, Commercial and Institutional (ICI)	Construction, Renovation, and Demolition (CRD)
Ville de Montréal-Est	1539	3840	6
Ville de Montréal	437607	546104	89146
Ville de Westmount	4749	533	53
Ville de Montréal-Ouest	1297	3006	5
Ville de Côte-Saint-Luc	7962	2871	22
Ville de Hampstead	1886	28	153
Ville de Mont-Royal	5107	3973	219
Ville de Dorval	4400	14130	1839
Ville de Pointe-Claire	5725	7366	607
Ville de Kirkland	4230	2549	890
Ville de Beaconsfield	3574	609	735
Ville de Baie-D'Urfé	1189	5777	691
Ville de Sainte-Anne-de-Bellevue	1300	970	285
Village de Senneville	219	479	231
Ville de Dollard-des-Ormeaux	13355	6870	838
Montreal Island total	494 138	599 102	95 721

Table S15. Emission factor for CO_2 in wastewater treatment

**Emission factor (kg CO_2/unit)**	**Unit**	**Area**	**Reference**
0.5	M^3		(Gupta and Singh, 2012)
0.03	kg	India	
0.26	kg CO_2-eq /day/EP		(Gupta and Singh, 2012)

Table S16. Emission factor for N_2O and CH_4 in wastewater treatment

**EF(g/Unit)**	**Gas type**	**Unit**	**Area**	**Reference**
0.15	CH_4	kg BOD	Denmark	(National Environmental Research Institute, 2005)
0.03	CH_4	kg BOD	Noida	(Gupta and Singh, 2012)
0.26	CH_4	kg BOD		(National Environmental Research Institute, 2005)
0.15	CH_4	kg BOD		(National Environmental Research Institute, 2005)
0.65	CH_4	kg BOD		(Gupta and Singh, 2012)
0.80	CH_4	kg CO_2-eq /day/EP		(Listowski et al., 2011)
0.007	N_2O	person per year	Germany	(National Environmental Research Institute, 2005)
0.0032	N_2O	person per year	Netherland	(National Environmental Research Institute, 2005)
1.57	N_2O	kg N		(Gupta and Singh, 2012)
0.30	N_2O	kg CO_2-eq /day/EP	Noida	(Listowski et al., 2011)

In city greenhouse gas assessments, it's important to account for landscape carbon sequestration because urban greenery helps absorb CO_2. This sequestration is typically calculated by multiplying the total number of trees in the city's landscape by the carbon sequestration factor of each tree. However, it's worth noting that this factor can vary depending on the age of the trees. In our study, we've used an average value for this factor to simplify the calculation process.

Table S17. Global warming potential (GHG emissions protocol, 2016)

**Species**	**Chemical Formula**	**Global Warming Potential**
Carbon dioxide	CO_2	1
Methane	CH_4	25
Nitrous oxide	N_2O	298

Table S18. Alias relationships for 2^{10-4} fractional factorial analysis about emission factors

**Factor**	**Definition**	**Low Level (-1)**	**High Level (+1)**
[A]	Emission factor of heating fuel	1.888	1.926
[B]	Emission factor of the energy (Gasoline)	55.44	83.16
[C]	Emission factor of the energy (Diesel)	59.26	88.88
[D]	Emission factor of the energy (Biodiesel)	57.14	85.7
[E]	Emission factor of suburban public transportation type	0.01216	0.01824
[F]	Emission factor of fuel consumption (Light vehicles)	1.76	2.64
[G]	Emission factor of fuel consumption (medium and Heavy vehicles)	2.064	3.096
[H]	Emission factor for CH_4 in wastewater	0.03	0.26
[J]	Emission factor for N_2O in wastewater	0.0004	0.0006
[K]	Emission factor for electricity generated by oil-fired power plant	676000	1014000

Table S19. Matrix for 2^{10-4} fractional factorial design regarding emission factors

EF_HEAT	EF_ENERGY (Gasoline)	EF_ENERGY (Diesel)	EF_ENERGY (Biodiesel)	EF_SPTN	EF_FUEL (Light vehicle)	EF_FUEL (Medium & Heavy vehicle)	EF_ CH_4	EF_ N_2O	EF_EGS (Thermal power)
1	-1	-1	-1	-1	1	-1	1	-1	-1
1	-1	1	1	-1	1	-1	1	1	1
-1	-1	1	-1	-1	1	1	1	1	-1
1	1	1	1	1	1	1	1	1	1
1	1	-1	1	1	1	-1	-1	1	-1
-1	-1	-1	1	-1	-1	-1	-1	-1	1
-1	-1	1	1	1	1	-1	-1	1	1
1	1	-1	-1	-1	1	1	1	1	1
1	1	1	-1	-1	1	-1	-1	1	-1
-1	-1	-1	-1	-1	-1	1	1	1	1
-1	1	-1	-1	1	-1	-1	1	1	1
1	1	-1	-1	-1	-1	-1	-1	1	1
-1	-1	-1	1	1	1	1	1	1	-1
1	1	-1	-1	1	-1	-1	-1	-1	-1
-1	-1	1	-1	-1	-1	-1	-1	1	-1
1	-1	1	-1	-1	1	1	-1	-1	1
1	-1	1	-1	1	-1	-1	1	1	-1
-1	1	1	1	1	-1	-1	1	-1	-1
1	1	-1	1	-1	-1	1	1	-1	1
-1	1	1	1	-1	1	1	-1	1	1
1	-1	-1	-1	1	-1	1	-1	1	1
-1	1	-1	-1	-1	-1	-1	1	-1	-1
1	1	1	1	-1	-1	-1	-1	-1	-1

EF_HEAT	EF_ENERGY (Gasoline)	EF_ENERGY (Diesel)	EF_ENERGY (Biodiesel)	EF_SPTN	EF_FUEL (Light vehicle)	EF_FUEL (Medium & Heavy vehicle)	EF_ CH_4	EF_ N_2O	EF_EGS (Thermal power)
1	-1	-1	-1	1	1	-1	1	1	1
1	1	1	1	1	-1	-1	-1	1	1
1	-1	-1	1	-1	-1	-1	1	1	-1
1	-1	-1	1	1	1	1	-1	-1	1
-1	1	1	-1	1	1	-1	1	1	-1
-1	1	-1	1	-1	1	-1	1	1	-1
-1	-1	1	-1	1	1	1	1	-1	1
1	1	-1	-1	1	1	1	1	-1	-1
1	-1	1	-1	1	1	1	-1	1	-1
-1	1	1	-1	1	-1	1	-1	1	-1
-1	-1	-1	-1	1	-1	1	1	-1	-1
-1	-1	1	1	-1	1	-1	-1	-1	-1
1	1	-1	1	1	-1	1	1	1	-1
-1	1	-1	1	1	1	-1	1	-1	1
-1	1	1	-1	-1	1	-1	1	-1	1
-1	-1	1	-1	1	-1	-1	-1	-1	1
-1	-1	-1	1	-1	1	1	1	-1	1
1	1	1	-1	1	1	-1	-1	-1	1
1	-1	-1	-1	-1	-1	1	-1	-1	-1
-1	1	-1	1	1	-1	1	-1	-1	1
1	1	1	-1	1	-1	1	1	-1	1
1	-1	1	1	-1	-1	1	-1	1	1
-1	-1	-1	-1	1	1	-1	-1	-1	-1
1	-1	-1	1	-1	1	1	-1	1	-1

EF_HEAT	EF_ENERGY (Gasoline)	EF_ENERGY (Diesel)	EF_ENERGY (Biodiesel)	EF_SPTN	EF_FUEL (Light vehicle)	EF_FUEL (Medium & Heavy vehicle)	EF_ CH_4	EF_ N_2O	EF_EGS (Thermal power)
-1	1	-1	-1	-1	1	1	-1	-1	-1
1	1	1	1	-1	1	1	1	-1	-1
-1	-1	-1	1	1	-1	-1	-1	1	-1
1	1	1	-1	-1	-1	1	1	1	-1
-1	-1	1	1	1	-1	1	1	1	1
1	-1	1	-1	-1	-1	-1	1	-1	1
-1	1	1	1	1	1	1	-1	-1	-1
1	1	-1	1	-1	1	-1	-1	-1	1
-1	-1	1	1	-1	-1	1	1	-1	-1
1	-1	1	1	1	-1	1	-1	-1	-1
1	-1	-1	1	1	-1	-1	1	-1	1
-1	1	-1	1	-1	-1	1	-1	1	-1
-1	1	-1	-1	1	1	1	-1	1	1
-1	-1	-1	-1	-1	1	-1	-1	1	1
-1	1	1	1	-1	-1	-1	1	1	1
1	-1	1	1	1	1	-1	1	-1	-1
-1	1	1	-1	-1	-1	1	-1	-1	1

Table S20. Alias relationships for 2^{10-4} fractional factorial analysis about other factors

**Factor**	**Definition**	**Low Level (-1)**	**High Level (+1)**
**[A]**	Total travel distance of suburban public transportation vessel	42157.5	183806.7
**[B]**	Average weight of suburban public transportation vessel	94.332	141.496
**[C]**	Average travel distance for light vehicles	11840	17760
**[D]**	Average travel distance for heavy vehicles	72000	108000
**[E]**	Degradable organic carbon in solid waste	0.12	0.18
**[F]**	Fraction of degradable organic carbon dissimilated	0.616	0.924
**[G]**	Concentration of BOD_5 in wastewater	0.00028	0.00042
**[H]**	Concentration of Nitrogen in raw wastewater	0.000048	0.000072
**[J]**	Ratio of electricity generation from different sources	0.01	0.99
**[K]**	Oil consumption	18	108

Table S21. Matrix for 2^{10-4} fractional factorial design regarding the other factors

TTD	AW	ATD_VEH (Light)	ATD_VEH (Heavy)	DOC_SW	F_DOC	CBOD	CN	RATIO_ELEC	Oil consumption
1	-1	1	-1	1	-1	-1	1	1	-1
1	-1	1	1	-1	1	-1	1	1	1
-1	1	-1	1	1	-1	1	-1	-1	1
-1	-1	-1	-1	1	1	-1	-1	-1	-1
1	1	-1	-1	1	1	1	1	-1	-1
1	-1	1	-1	-1	1	1	-1	-1	1
-1	-1	1	-1	1	-1	-1	-1	-1	1
1	1	1	1	-1	1	1	1	-1	-1
1	-1	1	-1	-1	-1	-1	1	-1	1
-1	1	-1	1	1	1	-1	1	-1	1
-1	-1	-1	-1	1	-1	1	1	-1	-1
-1	1	1	-1	-1	-1	1	-1	-1	1
-1	1	-1	1	-1	-1	1	-1	1	-1
-1	1	-1	-1	1	1	1	-1	1	1
-1	-1	-1	1	-1	1	1	1	-1	1
1	-1	-1	1	1	-1	-1	1	-1	1
1	1	1	-1	-1	1	-1	-1	1	-1
1	1	1	-1	1	-1	1	1	-1	1
-1	1	-1	-1	-1	-1	-1	1	-1	-1
1	-1	-1	1	-1	-1	-1	1	1	-1
-1	-1	-1	1	-1	-1	-1	-1	-1	1
1	1	-1	1	-1	1	-1	-1	-1	1
1	1	-1	-1	-1	-1	-1	-1	1	1
1	1	-1	1	-1	-1	1	1	-1	1

TTD	AW	ATD_VEH (Light)	ATD_VEH (Heavy)	DOC_SW	F_DOC	CBOD	CN	RATIO_ELEC	Oil consumption
-1	-1	1	1	-1	-1	1	1	-1	-1
1	1	1	-1	-1	-1	1	1	1	-1
-1	-1	1	-1	-1	1	1	1	1	-1
-1	1	-1	-1	1	-1	-1	1	1	1
-1	-1	1	-1	-1	-1	-1	-1	1	-1
-1	-1	-1	1	1	-1	-1	-1	1	-1
-1	-1	1	1	-1	1	-1	-1	-1	-1
1	-1	-1	-1	1	1	-1	1	1	1
1	1	1	1	1	1	1	1	1	1
1	1	-1	1	1	-1	1	1	1	-1
1	1	-1	-1	-1	1	1	1	1	1
1	1	1	-1	1	1	-1	-1	-1	1
-1	1	-1	1	-1	1	-1	1	1	-1
1	1	-1	1	1	1	-1	-1	1	-1
-1	1	-1	-1	-1	1	1	-1	-1	-1
-1	-1	1	1	1	-1	1	1	1	1
-1	-1	1	-1	1	1	1	1	-1	1
-1	-1	-1	-1	-1	1	-1	-1	1	1
-1	1	1	-1	1	1	-1	1	1	-1
1	-1	-1	1	1	1	1	-1	-1	1
-1	-1	-1	1	1	1	1	1	1	-1
1	1	1	1	-1	-1	-1	-1	-1	-1
1	-1	1	1	1	-1	1	-1	-1	-1
-1	-1	-1	-1	-1	-1	1	1	1	1
1	1	-1	-1	1	-1	-1	-1	-1	-1
1	-1	-1	-1	-1	1	-1	1	-1	-1
-1	1	1	1	-1	-1	-1	1	1	1

TTD	AW	ATD_VEH (Light)	ATD_VEH (Heavy)	DOC_SW	F_DOC	CBOD	CN	RATIO_ELEC	Oil consumption
-1	1	1	-1	1	-1	1	-1	1	-1
1	-1	1	1	-1	-1	1	-1	1	1
1	-1	1	-1	1	1	1	-1	1	-1
1	-1	1	1	1	1	-1	1	-1	-1
-1	1	1	1	-1	1	1	-1	1	1
-1	1	1	1	1	1	1	-1	-1	-1
1	1	1	1	1	-1	-1	-1	1	1
-1	1	1	1	1	-1	-1	1	-1	-1
1	-1	-1	-1	1	-1	1	-1	1	1
1	-1	-1	-1	-1	-1	1	-1	-1	-1
-1	1	1	-1	-1	1	-1	1	-1	1
1	-1	-1	1	-1	1	1	-1	1	-1
-1	-1	1	1	1	1	-1	-1	1	1

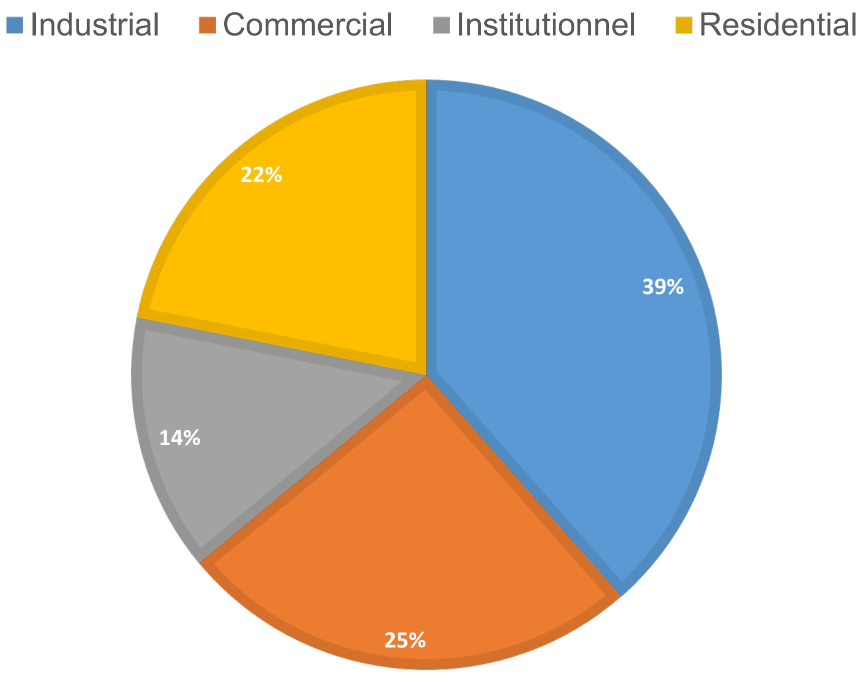

Figure S1. Natural gas distribution in Montreal by sector in 2016.

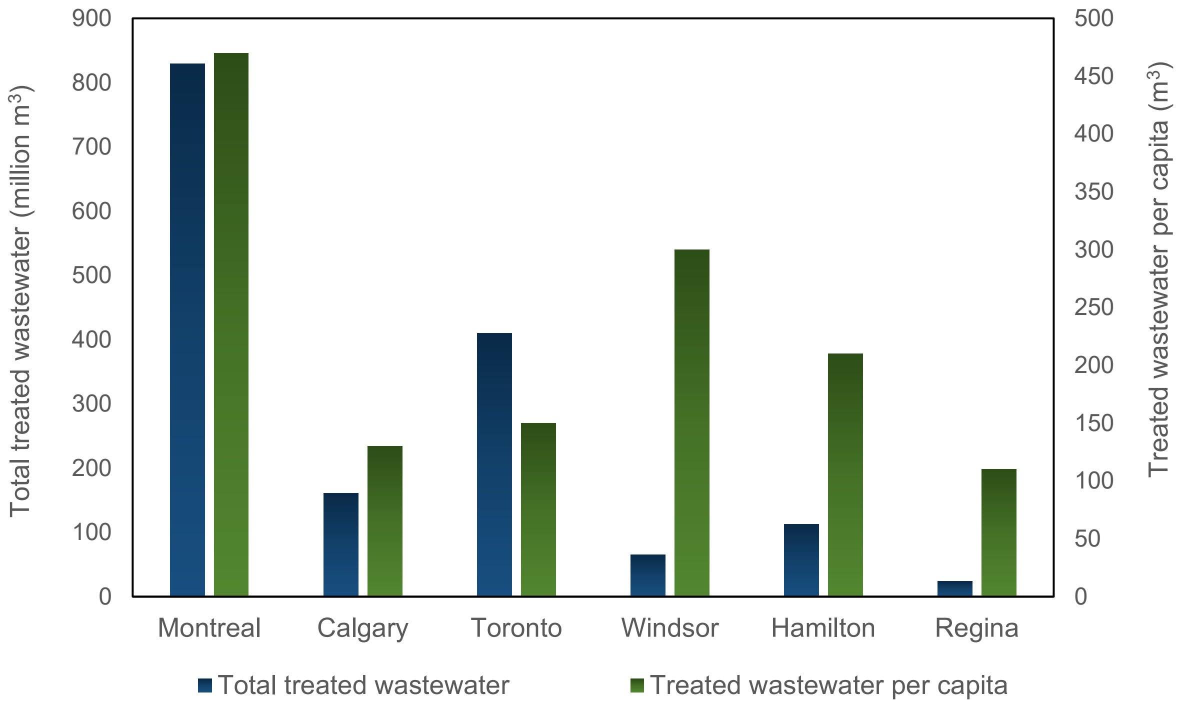

Figure S2 Treated wastewater in 2016.

References

Agência Portuguesa do Ambiente (2017) Portuguese national inventory report on greenhouse gases, 1990-2015. https://apambiente.pt/_zdata/Inventario/2017/20170530/NIRglobal20170526.pdf.

Australian Transport Assessment and Planning (ATAP) (2016) Australian transport assessment and planning guidelines, p. 69. https://www.atap.gov.au/.

Energir 2019 Natural gas consumption.

Environment Quebec (2018) Données d'élimination des matières résiduelles au Québec. http://www.environnement.gouv.qc.ca/matieres/donnees-elimination.htm.

GHG emissions protocol (2016) Global warming potential values https://www.ghgprotocol.org/sites/default/files/ghgp/Global-Warming-Potential-Values%20%28Feb%2016%202016%29_1.pdf.

Government of Canada (2017) National Inventory Report 1990–2017: Greenhouse Gas Sources and Sinks in Canada.

Gupta, D. and Singh, S.K. (2012) Greenhouse gas emissions from wastewater treatment plants: a case study of Noida. Journal of Water Sustainability 2(2), 131-139.

Hydro-Quebec (2012) Annual report 2012. http://www.hydroquebec.com/data/documents-donnees/pdf/annual-report-2012.pdf.

Hydro-Quebec (2014) Annual report 2014. http://www.hydroquebec.com/data/documents-donnees/pdf/annual-report-2014.pdf.

Hydro-Quebec (2016) Annual report 2016. http://www.hydroquebec.com/data/documents-donnees/pdf/annual-report-2016.pdf.

Koffi, B., Cerutti, A., Duerr, M., Iancu, A., Kona, A. and Janssens-Maenhout, G. (2017) Covenant of Mayors for climate and energy: default emission factors for local emission inventories. Publ. Off. Eur. Union.

Listowski, A., Ngo, H., Guo, W., Vigneswaran, S., Shin, H. and Moon, H. (2011) Greenhouse gas (GHG) emissions from urban wastewater system: future assessment framework and methodology. Journal of Water Sustainability 1(1), 113-125.

Ministry of Environment British Columbia (2016) Best practices methodology for quantifying greenhouse gas emissions.

National Environmental Research Institute (2005) Emission of CH4 and N2O from wastewater treatment plants.

Prakash, R. and Bhat, I. (2012) Life cycle greenhouse gas emissions estimation for small hydropower schemes in India. Energy 44(1), 498-508.

SenterNovem (2005) The Netherlands: list of fuels and standard CO2 emission factors.

Societe de Anssurance Automobile Quebec (2007) Dossier statistique bilan 2007. https://saaq.gouv.qc.ca/fileadmin/documents/publications/espace-recherche/dossier-statistique-bilan-2007.pdf.

Societe de Anssurance Automobile Quebec (2008) Dossier statistique bilan 2008. https://saaq.gouv.qc.ca/fileadmin/documents/publications/espace-recherche/dossier-statistique-bilan-2008.pdf.

Societe de Anssurance Automobile Quebec (2009) Dossier statistique bilan 2009. https://saaq.gouv.qc.ca/fileadmin/documents/publications/espace-recherche/dossier-statistique-bilan-2009.pdf.

Societe de Anssurance Automobile Quebec (2016) Dossier statistique bilan 2016.

Societe de Transport de Montreal STM (2016) Sustainable development report 2016. http://www.stm.info/sites/default/files/pdf/en/a-rdd2016.pdf.

City of New York (2017) Inventory of New York City greenhouse gas emissions in 2016.

U.S. EPA (2018) Emission Factors for Greenhouse Gas Inventories.

William Steinhurst, P.K. and Schultz, a.M. (2012) Hydropower GHG emission.

Zhang, Q.F., Karney, B., MacLean, H.L. and Feng, J.C. (2007) Life-cycle inventory of energy use and greenhouse gas emissions for two hydropower projects in China. Journal of Infrastructure Systems 13(4), 271-279.
